# Novel solid-phase strategy for the synthesis of ligand-targeted fluorescent-labelled chelating peptide conjugates as a theranostic tool for cancer

**DOI:** 10.3762/bjoc.14.244

**Published:** 2018-10-18

**Authors:** Sagnik Sengupta, Mena Asha Krishnan, Premansh Dudhe, Ramesh B Reddy, Bishnubasu Giri, Sudeshna Chattopadhyay, Venkatesh Chelvam

**Affiliations:** 1 Discipline of Chemistry, Indian Institute of Technology Indore, Khandwa Road, Simrol, Indore 453 552, India; 2 Discipline of Biosciences and Biomedical Engineering, Indian Institute of Technology Indore, Khandwa Road, Simrol, Indore 453 552, India; 3 Discipline of Physics and Discipline of Metallurgy Engineering & Material Sciences, Indian Institute of Technology Indore, Khandwa Road, Simrol, Indore 453 552, India

**Keywords:** chelating linker, confocal studies, continuous synthetic process, fluorescent tag, Fmoc-Lys-(Tfa)-OH, prostate cancer and ovarian cancer, solid-phase peptide synthesis

## Abstract

In this article, we have successfully designed and demonstrated a novel continuous process for assembling targeting ligands, peptidic spacers, fluorescent tags and a chelating core for the attachment of cytotoxic molecules, radiotracers, nanomaterials in a standard Fmoc solid-phase peptide synthesis in high yield and purity. The differentially protected Fmoc-Lys-(Tfa)-OH plays a vital role in attaching fluorescent tags while growing the peptide chain in an uninterrupted manner. The methodology is versatile for solid-phase resins that are sensitive to mild and strong acidic conditions when acid-sensitive side chain amino protecting groups such as Trt (chlorotrityl), Mtt (4-methyltrityl), Mmt (4-methoxytrityl) are employed to synthesise the ligand targeted fluorescent tagged bioconjugates. Using this methodology, DUPA rhodamine B conjugate (DUPA = 2-[3-(1,3-dicarboxypropyl)ureido]pentanedioic acid), targeting prostate specific membrane antigen (PSMA) expressed on prostate, breast, bladder and brain cancers and pteroate rhodamine B, targeting folate receptor positive cancers such as ovarian, lung, endometrium as well as inflammatory diseases have been synthesized. In vitro studies using LNCaP (PSMA +ve), PC-3 (PSMA −ve, FR −ve) and CHO-β (FR +ve) cell lines and their respective competition experiments demonstrate the specificity of the newly synthesized bioconstructs for future application in fluorescent guided intra-operative imaging.

## Introduction

The understanding of cell processes is indispensable to devise new strategies for diagnosis and treatment of cancer and inflammatory diseases through targeted drug delivery techniques [1]. The complex molecular processes in a cell are discerned by tagging fluorescent probes or radioactive tracers to a targeting ligand that will undergo internalization after binding to cell surface proteins overexpressed in diseased conditions. The internalized tracers along with the targeting ligand act as a tracking molecule to understand the destination of delivered cargos or biologics. For bioimaging of cancer and inflammatory diseases through specific biomarkers [2], several methods including single positron emission computed tomography (SPECT), positron emission tomography (PET) and magnetic resonance imaging (MRI) are exploited and each modality has its own strengths and weaknesses [3]. However, imaging studies using fluorescent probes [4] or radioactive isotopes [5–6] offers real-time, non-invasive, high-resolution images, during the examination of pathological diseased state.

Prostate specific membrane antigen (PSMA) [7–9] and the folate receptor [10–13] are well characterized and most attractive cancer biomarkers present in primary and metastatic stages of prostate and ovarian cancers, respectively. PSMA belongs to a family of type II membrane bound glycoprotein over-expressed on the cell surface of prostate, brain, bladder and breast cancers. Whereas folate receptors are attached to the cell membrane by a glycophosphatidylinositol anchor and over-expressed on several cancers as well as activated macrophages during inflammation. Moreover, folate receptors were also discovered to be overexpressed on activated macrophages [14] but not on resting macrophages [15]. Many inflammatory diseases such as rheumatoid arthritis, inflammatory osteoarthritis, ischemia reperfusion injury, atherosclerosis, psoriasis, vasculitis, lupus, diabetes, glomerulonephritis, sarcoidosis, Crohn’s and Sjogren's disease are caused by activated macrophages [16]. Recently EC17, (λ_ex_ = 465–490 nm and λ_em_ = 520–530 nm) a conjugate of folic acid and fluorescein isothiocyanate has been used for intraoperative surgery of ovarian cancer [17], lung adenocarcinoma [18–20], and breast cancer [21]. Therefore, targeting these biomarkers brings forth new insight to know the cause and treatment for such ailments. These biomarkers belong to a family of cell surface transmembrane proteins [22] over-expressed mainly in diseased tissues and exploited in delivering chemical tools for early diagnosis of malignancy [23] and inflammatory diseases. They are also utilized for targeted drug delivery [24–25] of therapeutics to avoid any off-site toxicity to normal and healthy cells. Unfortunately, strategies to construct diagnostic and therapeutic chemical tools consisting of a polypeptidic spacer, a homing ligand for biomarkers, a fluorescent tag and a chelating moiety for tethering cargo in a continuous process using solid-phase peptide synthesis is poorly developed. Traditional solid-phase peptide synthesis methods for preparation of bioconstructs employ orthogonally protected functional moieties present in commercial resins such as Universal Nova tag or hyperacid labile resins such as Rink acid [26], 4-hydroxymethylphenoxybutyryl (HMPB), chlorotrityl [27], SASRIN [28] and Sieber amide [29]. Even though such resins are very useful, they suffer from several disadvantages. For example, i) they are cost ineffective, ii) possess low resin loading, iii) incompatible in medium to strongly acidic [30] or basic conditions employed for deprotection of coupled amino acids and iv) undergo premature cleavage of polypeptide chain from solid support resulting in moderate yield during deprotection of acid sensitive side chain protecting moieties to introduce fluorescent tags.

**Figure 1:**
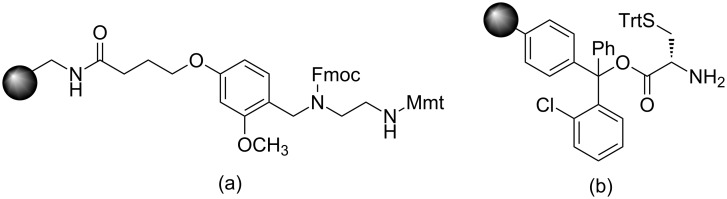
(a) Structure of universal nova tag resin, (b) structure of H-L-Cys(Trt)-2-ClTrt resin.

In addition to the above drawbacks, conventional methods for the synthesis of targeted fluorescent tagged bioconjugates [31] are a mixed approach of both solid and solution phase synthesis [32]. These involve several intermediary purification steps to separate side products and unreacted fluorescent components. Moreover, there are reports wherein receptor-targeted multimodal tools have been synthesized solely by employing solution phase chemistry [33–35]. These multistep synthetic protocol results in the escalation of the cost of intra-operative imaging tools that would otherwise be produced by our methodology with a single purification step. Even though Universal Nova tag resin [36] (Figure 1a) has resolved this problem to some extent, it suffers from a problem of employing acidic condition to deprotect side chain 4-methoxytrityl (Mmt) amino protecting group before attachment of fluorescent tag with the peptidic spacer. This results in premature cleavage of the peptide chain and loss of chemical yield during the bioconjugate synthesis. Further, attaching a radiotracer chelating core containing acid sensitive functional groups and the amino acid cysteine is also cumbersome and challenging. Recently, Low et al. reported synthesis of various targeted conjugates in which fluorescent tag [37] has been attached in a solution phase reaction. Also, they have reported the synthesis of ligand-conjugated peptides containing a radiotracer segment [38] without fluorescent tag using Wang resin that is cleaved in strongly acidic conditions.

Contrary to the aforementioned drawbacks, the present manuscript elicits a novel synthetic strategy for building new bioconstructs with several components in a continuous process without isolation of any of the intermediates. The various components that are assembled include the cell surface protein recognition ligand, a peptide spacer for enhanced solubility and binding affinity, a fluorescent tag for tissue staining and a chelating core to tether therapeutic cargos. This goal is smoothly achieved in high chemical yield and purity by strategically introducing differentially protected dibasic amino acids such as lysine whose α- and ε-amino groups are protected as base labile Fmoc and trifluoroacetyl (Tfa) protecting groups, respectively. The whole concept is successfully illustrated using commonly available and less expensive cysteine-labelled 2-chlorotrityl resin (Figure 1b). The methodology is general and can be significantly useful for acid-sensitive resins that contain acid-labile orthogonal amino acids with 4-methoxytrityl (Mmt) and 4-methyltrityl (Mtt) protecting groups.

## Results and Discussion

**Figure 2:**
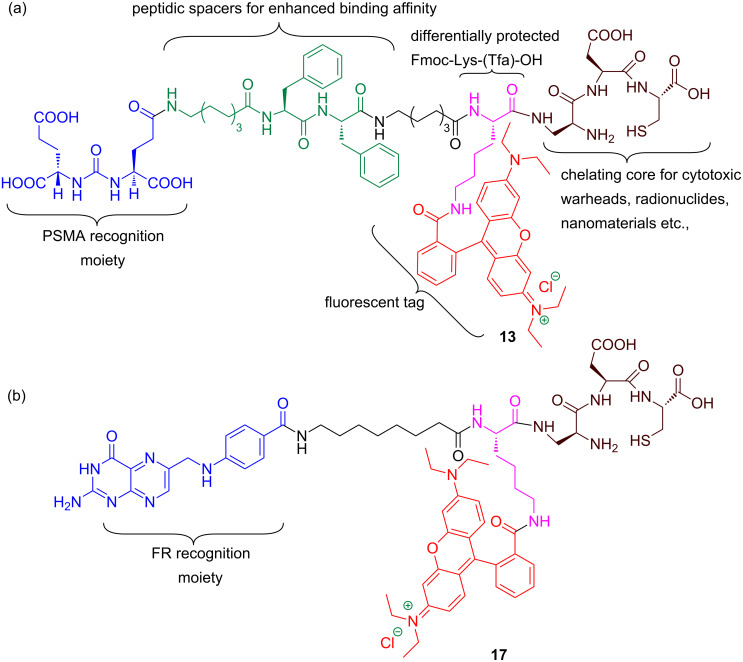
(a) PSMA targeted DUPA rhodamine B chelating conjugate **13**. (b) Folate receptor targeted pteroate rhodamine B chelating conjugate **17**.

PSMA has a very high affinity [39] for a small molecule homing ligand called DUPA or 2-[3-(1,3-dicarboxypropyl)ureido]pentanedioic acid with an inhibition constant *K*_i_ of 8 nM. Folate protein binds to folic acid and their derivatives [40] such as pteroate ligand [41] with high degree of specificity (*K*_d_ ≈ 10 nM) to deliver attached cargos to the interior of cells. These targeting ligands, DUPA and pteroate, have been exploited in the design and synthesis of our new ligand targeted tracer conjugates **13** and **17** (Figure 2) to target PSMA^+^ and FR^+^ diseased conditions.

Analysis of the crystal structure [42] of PSMA reveals that small molecule ligands such as DUPA would reach the PSMA active site through a gradually narrowing tunnel of amino acids of 20 Å length. Moreover, the inner surface of the PSMA tunnel possesses two hydrophobic pockets suitable for hydrophobic interactions with the amino acids present in the peptide spacer [38]. Therefore, it is pertinent to design a PSMA targeted conjugate that can pass through the tunnel smoothly and reach the active site as well as fit in hydrophobic pockets via hydrophobic interactions. Additionally, the carbonyl oxygen of the urea moiety of DUPA directly coordinates with two zinc atoms present in the active site of PSMA. The γ′-carboxylic acid of the DUPA ligand does not play a significant role in the interaction with the PSMA active site and hence exploited as a handle for the construction of peptidic spacer of bioconjugate **13**.

While designing the required peptide spacer [38] of **13** (Figure 2) for the tunnel, an eight-carbon amino acid such as 8-aminocaprylic acid has been covalently attached to the γ′-carboxylic acid of the DUPA ligand. This ensures the adequate distance between targeting ligand and peptidic spacer so that the specific binding to the protein is not compromised. The additional distance and hydrophobic pockets present in the 20 Å channel is crossed over by introduction of two phenylalanine (Phe) amino acids in the spacer. The polypeptide chain is also attached to another molecule of 8-aminocaprylic acid to ensure that the molecular position of the fluorescent tag and chelating core would lie outside the surface of the protein tunnel. Moreover, differentially protected α- and ε-amino groups of the amino acid lysine (Lys), Fmoc-Lys-(Tfa)-OH, is also introduced in the peptide chain. The main purpose of this exercise is to connect the chelating core through carboxylic acid of lysine and attachment of fluorescent probe via ε-amino group present in the lysine amino acid. Increased hydrophobicity due to the introduction of long chain amino acids, aromatic amino acids in the targeted ligand peptide conjugate **13** would decrease the solubility. This is compensated by introduction of dibasic amino acid like diaminopropionic acid (Dap), acidic amino acids like aspartic acid (Asp) and polar cysteine amino acid (Cys) that makes up the chelating core.

In the case of FR targeted fluorescent conjugate **17**, the targeting ligand, folic acid, is modified by removal of the L-glutamic acid residue to give the pteroic acid moiety (Figure 2). The binding affinity of the modified folate is relatively weaker than folic acid [41]. The targeting ligand, pteroic acid, is covalently coupled to 8-aminocaprylic acid to separate the active binding site of folate protein from the interference of fluorescent cargo attached to lysine and the chelating core as described for **13**.

Thus our newly designed bioconstructs **13** and **17** have the following four components, (i) a cell surface protein recognition ligand, (ii) a peptidic spacer which enhances the binding affinity of the PSMA-targeting conjugate **13**; it minimizes the repulsive interaction between the bulky dye molecule and the targeted folate protein active site in the case of folate receptor targeting bioconstruct **17**, (iii) a fluorescent tag to track the cellular destination of bioconjugates and visualization aid for tissue staining, (iv) a chelating core as a multipurpose handle for loading drug cargos, radionuclides, or nanomaterials.

**Scheme 1:**
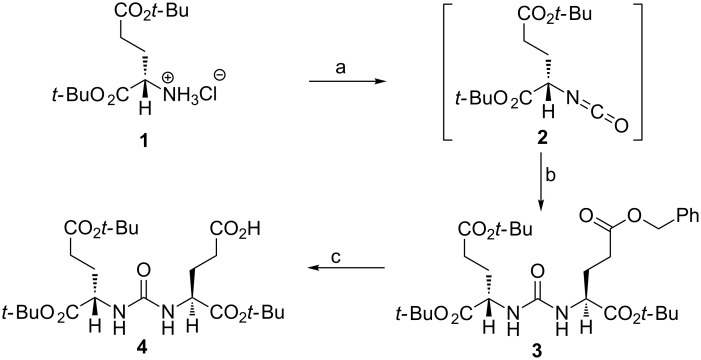
Synthesis of PSMA tris(*tert*-butoxy) protected DUPA ligand **4**. Reagents and conditions: (a) Triphosgene, triethylamine, dichloromethane (DCM), −50 °C to rt; (b) L-glutamic acid γ-benzyl-α-*tert*-butylester hydrochloride, triethylamine, DCM, rt, overnight; (c) H_2_, Pd/C, CH_2_Cl_2_, 24 h, rt.

The tris(*tert*-butoxy) protected DUPA precursor **4** required for the preparation of conjugate **13** was prepared as per reported procedure [38] (Scheme 1).

**Scheme 2:**
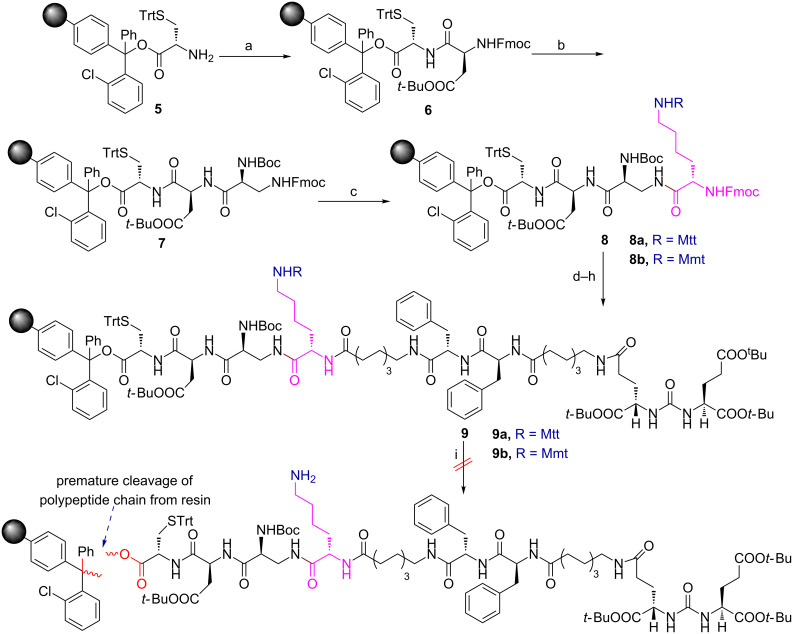
Attempted synthesis of PSMA targeted DUPA rhodamine B chelating conjugate **13** using Fmoc-Lys(Mtt/Mmt)-OH. Reagents and conditions: (a) Fmoc-Asp(O*t-*Bu)-OH, PyBOP, DIPEA, DMF, 6 h; (b) (1) 20% piperidine in DMF, rt, 30 min; (2) Boc-Dap(Fmoc)-OH, PyBOP, DIPEA, DMF, 6 h; (c) (1) 20% piperidine in DMF, rt, 30 min; (2) Fmoc-Lys(Mtt/Mmt)-OH, PyBOP, DIPEA, DMF, 6 h; (d) (1) 20% piperidine in DMF, rt, 30 min; (2) Fmoc-8-aminocaprylic acid, PyBOP, DIPEA, DMF, 6 h; (e) (1) 20% piperidine in DMF, rt, 30 min; (2) Fmoc-Phe-OH, PyBOP, DIPEA, DMF, 6 h; (f) (1) 20% piperidine in DMF, rt, 30 min; (2) Fmoc-Phe-OH, PyBOP, DIPEA, DMF, 6 h; (g) (1) 20% piperidine in DMF, rt, 30 min; (2) Fmoc-8-aminocaprylic acid, PyBOP, DIPEA, DMF, 6 h; (h) (1) 20% piperidine in DMF, rt, 30 min; (2) DUPA(O*t-*Bu)_3_-OH **4**, PyBOP, DIPEA, DMF, 6 h; (i) acetic acid/ trifluoroethanol/DCM (1:2:7), rt, 1 h or HOBt (1 M) in DCM/TFE (1:1), 1 h.

Starting from commercially available cysteine capped chlorotrityl resin, H-L-Cys(Trt)-2-ClTrt **5**, we begin the synthesis of bioconjugate **13** as shown in Scheme 2. Using standard Fmoc solid-phase peptide synthesis methodology, amino acids such as Fmoc-Asp(O*t*-Bu)-OH, Boc-Dap(Fmoc)-OH were coupled in sequence to cysteine amino acid attached to chlorotrityl resin via dipeptide intermediate **6** to give the tripeptide intermediate **7**. The tripeptide **7** was then attached to strategic lysine amino acid, Fmoc-Lys-(Pg)-OH, whose ε-amino group is protected as either an Mtt (4-methyltrityl) or an Mmt (4-methoxytrityl) protecting group (Pg) to give tetrapeptides **8a** or **8b**. The tetrapeptides **8a** or **8b** were tethered sequentially to 8-aminocaprylic acid, two phenyalanine residues, another 8-aminocaprylic acid and finally to DUPA precursor **4**, to provide polypeptide chains **9a** or **9b** (Scheme 2).

The ε-amino protecting groups present in the polypeptide chains **9a** (Pg = Mtt) and **9b** (Pg = Mmt) are generally cleaved under acidic conditions. Therefore, it becomes pertinent to analyze the stability of the chlorotrityl resin and ε-amino protecting groups in the polypeptide chains **9a** and **9b** to achieve our multiple objectives in a continuous synthetic process without the isolation of any of the intermediates.

The peptide chain cleavage conditions for chlorotrityl resin are well established and the ε-amino trityl protecting groups of Fmoc-Lys-(Mtt or Mmt)-OH of the side chain are usually acid-labile with an order of stability: Trt (chlorotrityl) > Mtt (4-methyltrityl) > Mmt (4-methoxytrityl). In our initial attempt, we opined that the Mtt- (4-methyltrityl-) protected ε-amino group of Fmoc-Lys-(Mtt)-OH should undergo selective cleavage under mildly acidic conditions without cleavage of polypeptide chain **9a** from the resin. Selective deprotection of the Mtt protecting group was achieved when **9a** was treated with either 1% TFA in dichloromethane or a mixture of acetic acid/trifluoroethanol/DCM in 1:2:7 ratio for 1 h at room temperature [43]. Unfortunately, the polypeptide chain **9a** cleaved off too from the resin beads (Scheme 2). Therefore, it became difficult to identify and marginally separate the acidic conditions required for selective cleavage of the Mtt protecting group in the side chain of **9a** from chlorotrityl resin.

Because of this reason we questioned the introduction of better electron-releasing groups such as 4-methoxytrityl (Mmt) instead of Mtt in the strategic amino acid, as in the case of Fmoc-Lys-(Mmt)-OH. This would increase the margin of difference and lower the acid strength required for the exclusive cleavage of the Mmt protecting group in the side chain of the **9b** from the chlorotrityl resin. With this view, **9b** containing Fmoc-Lys-(Mmt)-OH is subjected to cleavage under milder acidic conditions using 1 M HOBt (hydroxybenzotriazole) in trifluoroethanol/dichloromethane (1:1). However, these conditions did not provide the required solution and failed to differentiate the acidic strength needed for exclusive cleavage of the Mmt protecting group in the side chain of **9b** from the chlorotrityl resin resulting in the detachment of the polypeptide chain **9b** (Scheme 2). Therefore, we turned our attention to replace the acid labile trityl protecting groups with a base labile protecting group such as trifluoroacetyl (Tfa) as in the case of Fmoc-Lys-(Tfa)-OH amino acid.

**Scheme 3:**
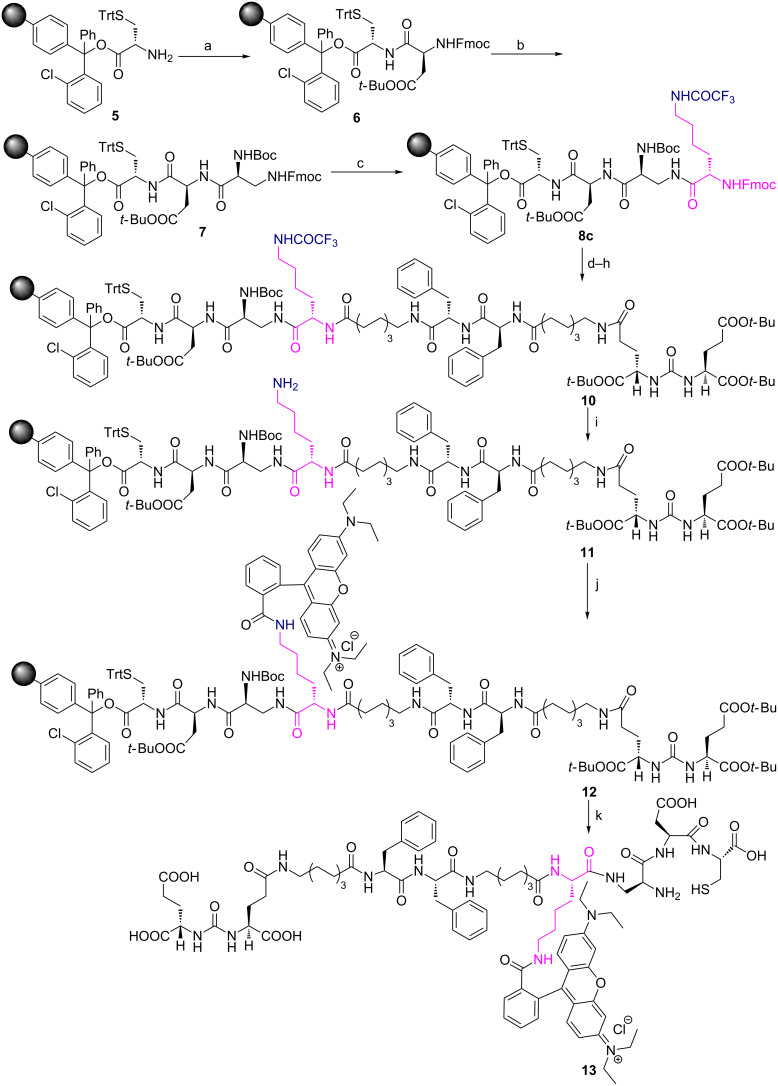
Synthesis of PSMA targeting DUPA rhodamine B chelating conjugate **13**. Reagents and conditions: (a) Fmoc-Asp(O*t*-Bu)-OH, PyBOP, DIPEA, DMF, 6 h; (b) (1) 20% piperidine in DMF, rt, 30 min; (2) Boc-Dap(Fmoc)-OH, PyBOP, DIPEA, DMF, 6 h; (c) (1) 20% piperidine in DMF, rt, 30 min; (2) Fmoc-Lys(Tfa)-OH, PyBOP, DIPEA, DMF, 6 h; (d) (1) 20% piperidine in DMF, rt, 30 min; (2) Fmoc-8-aminocaprylic acid, PyBOP, DIPEA, DMF, 6 h; (e) (1) 20% piperidine in DMF, rt, 30 min; (2) Fmoc-Phe-OH, PyBOP, DIPEA, DMF, 6 h; (f) (1) 20% piperidine in DMF, rt, 30 min; (2) Fmoc-Phe-OH, PyBOP, DIPEA, DMF, 6 h; (g) (1) 20% piperidine in DMF, rt, 30 min; (2) Fmoc-8-aminocaprylic acid, PyBOP, DIPEA, DMF, 6 h; (h) (1) 20% piperidine in DMF, rt, 30 min; (2) DUPA(O*t*-Bu)_3_-OH, PyBOP, DIPEA, DMF, 6 h; (i) 2 M piperidine in water, rt, 6–12 h; (j) rhodamine B, PyBOP, DIPEA, DMF, 6 h; (k) (1) TFA/TIS/EDT/H_2_O (9.25:0.25:0.25:0.25, 1 × 5 mL, 30 min; 2 × 5 mL, 5 min); (2) evaporate TFA; (3) precipitate in ice cold diethyl ether.

With the hope that introduction of a base-labile protecting group in lysine could provide the required solution, we began the synthesis of polypeptide chain **10** with Fmoc-Lys-(Tfa)-OH derivative (Scheme 3). This idea for the synthesis of polypeptide chain **10** has been adapted from an earlier work reported by Moroder et al*.* in which trifluoroacetyl (Tfa) moiety was deprotected during a peptide synthesis [44] using 1 M aqueous piperidine at ice cold temperature. The polypeptide chain **10** was thus subjected to 1 M aqueous piperidine at 0 °C to deprotect the ε-amino trifluoroacetyl moiety. The literature reported conditions, however, failed to deprotect the Tfa group from polypeptide chain **10**. We have tested few reaction conditions and after optimization, we have successfully deprotected ε-amino Tfa protecting group from the side chain using 2 M aq piperidine at room temperature for 6–12 h (the completion of the reaction being monitored through the Kaiser’s test) to give polypeptide chain **11** with free ε-amino group. It is also interesting to note that the reaction is smooth, clean without resulting in rupture of the polypeptide chain **10** from chlorotrityl resin. The protecting groups of other amino acids present in **11** remain intact during this process. After the successful cleavage of the Tfa protecting group to give **11**, the free ε-amino group was covalently bonded to a fluorescent tag such as rhodamine B using standard peptide coupling chemistry to afford rhodamine B conjugated polypeptide chain **12**. Amino acid protecting groups such as Boc, *tert*-butyl and Trt of diaminopropionic acid, aspartic acid and cysteine thiol moieties, respectively, in **12** were cleaved traceless using a cocktail of trifluoroacetic acid, triisopropylsilane, ethanedithiol in water. The final PSMA targeted rhodamine B peptide conjugate **13** was thus obtained from chlorotrityl resin as shown in Scheme 3 in high yield and purity. By using this new procedure, we have successfully demonstrated for the first-time a selective cleavage of the base sensitive ε-amino protecting group (Tfa) in the side chain of the growing polypeptide chain. The methodology could be extended to incorporate any basic natural or unnatural amino acids in a peptide chain in which the α-amino group is protected as Fmoc and the side chain amino group, in any position (β, γ, δ) along the carbon side chain, is protected as a trifluoroacetyl moiety. The methodology is thus successfully utilized to install fluorescent tags that are sensitive to strong inorganic bases. Moreover, the methodology is applicable to resins that are sensitive to mild and strong acidic conditions and peptide chains that contain side chain protecting groups such as Trt (chlorotrityl), Mtt (4-methyltrityl), Mmt (4-methoxytrityl) for the synthesis of targeted fluorescent bioconjugates.

**Scheme 4:**
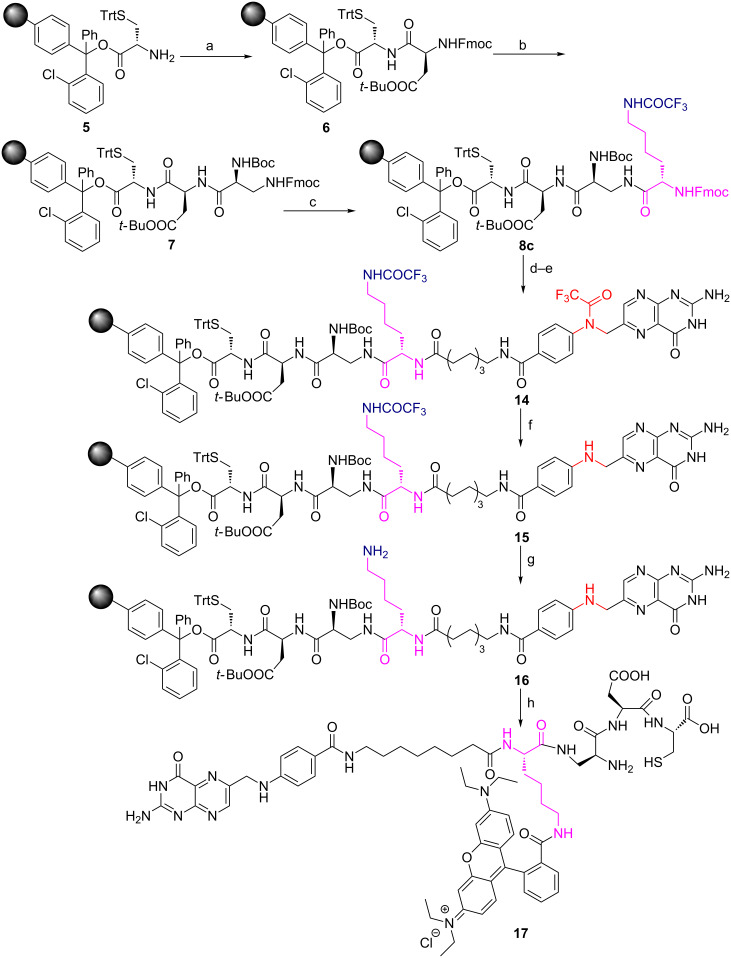
Synthesis of folate receptor targeting pteroate rhodamine B chelating conjugate **17**. Reagents and conditions: (a) Fmoc-Asp(O*t*-Bu)-OH, PyBOP, DIPEA, DMF, 6 h; (b) (1) 20% piperidine in DMF, rt, 30 min; (2) Boc-Dap(Fmoc)-OH, PyBOP, DIPEA, DMF, 6 h; (c) (1) 20% piperidine in DMF, rt, 30 min; (2) Fmoc-Lys(Tfa)-OH, PyBOP, DIPEA, DMF, 6 h; (d) (1) 20% piperidine in DMF, rt, 30 min; (2) Fmoc-8-aminocaprylic acid, PyBOP, DIPEA, DMF, 6 h; (e) (1) 20% piperidine in DMF, rt, 30 min; (2) *N*^10^-(trifluoroacetyl)pteroic acid, PyBop, HOBt·H_2_O, DIPEA, DMSO, DMF, 6 h; (f) 1% NH_2_NH_2_·H_2_O, DMF (3 × 2 mL) 10 min each; (g) 2 M piperidine in water, rt, 6–12 h; (h) (1) rhodamine B, PyBOP, DIPEA, DMF, 6 h; (2) TFA/TIS/EDT/H_2_O (9.25:0.25:0.25:0.25, 1 × 5 mL, 30 min; 2 × 5 mL, 5 min); (3) evaporate TFA; (4) precipitate in ice cold diethyl ether.

The methodology was further extended to synthesize FR targeted fluorescent chelating conjugate **17** as shown in Scheme 4. Using standard Fmoc SPPS methodology, Fmoc amino acids such as Fmoc-Asp(O*t*-Bu)-OH, Boc-Dap(Fmoc)-OH were coupled in sequence to STrt protected cysteine amino acid attached to a chlorotrityl resin to give dipeptide **6** and tripeptide **7** intermediates**.** The tripeptide **7** was then attached to Fmoc-Lys-(Tfa)-OH to give tetrapeptide **8c**. The tetrapeptide **8c** was coupled to Fmoc-8-aminocaprylic acid followed by the introduction of folate protein targeting ligand in the form of *N*^10^-(trifluoroacetyl)pteroic acid to give polypeptide **14**. It’s noteworthy to mention that the ligand targeted chelating polypeptide **14** contains two different trifluoroacetyl moieties which protect the secondary amine of pteroate core as well as primary amine of ε-amino lysine residue. The *N*^10^-(trifluoroacetyl) or secondary amine protecting group of pteroic acid is selectively cleaved using 1% NH_2_NH_2_·H_2_O in DMF without affecting the trifluoroacetyl protecting group of lysine residue. This is unequivocally confirmed by performing the Kaiser test on polypeptide chain **15** which does not turn dark blue after the cleavage of the *N*^10^-(trifluoroacetyl) group from the pteroate entity (Scheme 4). The secondary amine of the pteroate core generated in **15** after the cleavage of the *N*^10^-(trifluoroacetyl) group does not give positive Kaiser test (see Experimental section).

Using 2 M aqueous piperidine we successfully cleaved the Tfa protecting group from the side chain of **15** to give a polypeptide chain **16** that is still intact with the resin. The successful cleavage of the Tfa group from the lysine side chain is confirmed by performing the Kaiser test on resin beads containing polypeptide chain **16** which turned dark blue. The free ε-amino group thus liberated was covalently attached to the fluorescent tag such as tetraethylrhodamine B by standard peptide bond formation chemistry. Protecting groups such as Boc, *tert*-butyl and Trt present in diaminopropionic acid, aspartic acid and cysteine thiol aminoacids, respectively, were cleaved using a cocktail of trifluoroacetic acid, triisopropyl silane, ethanedithiol in water to give folate receptor targeted chelating rhodamine B conjugate **17** in high yield and purity (Scheme 4).

Because bioconjugates **13** and **17** have the amino acid cysteine in the peptide chain, which is an essential requirement for construction of the chelating core, other protected ε-amino lysine derivatives [45–46] such as *N*-Fmoc-Lys(Alloc)-OH have not been employed in our synthetic strategy. Usually, the allyl protecting group in *N*-Fmoc-Lys(Alloc)-OH is deprotected using Pd(PPh_3_)_4_ catalyst. However, the sulfur atom present in the cysteine moiety of the chelating core is known to poison palladium catalysts resulting in complete failure of the deprotection of the ε-amino allyl protecting moiety. Moreover, palladium is a heavy metal and traceless removal of it from the resulting bioconjugates **13** and **17** is near to impossible. Since the prepared bioconjugates **13** and **17** are to be utilized for imaging of human cancer cell lines, presence of any heavy metals in the synthetic strategy would cause unwanted toxicity and inaccuracy in the biological study. The SH group present in the bioconjugate handle or chelating core of compound **13** and **17** can be utilized for the attachment of drugs [47], nanomaterial and radionuclide for therapeutic purposes. This added advantage makes the bioconjugates a potential theranostic tool for cancer.

**Figure 3:**
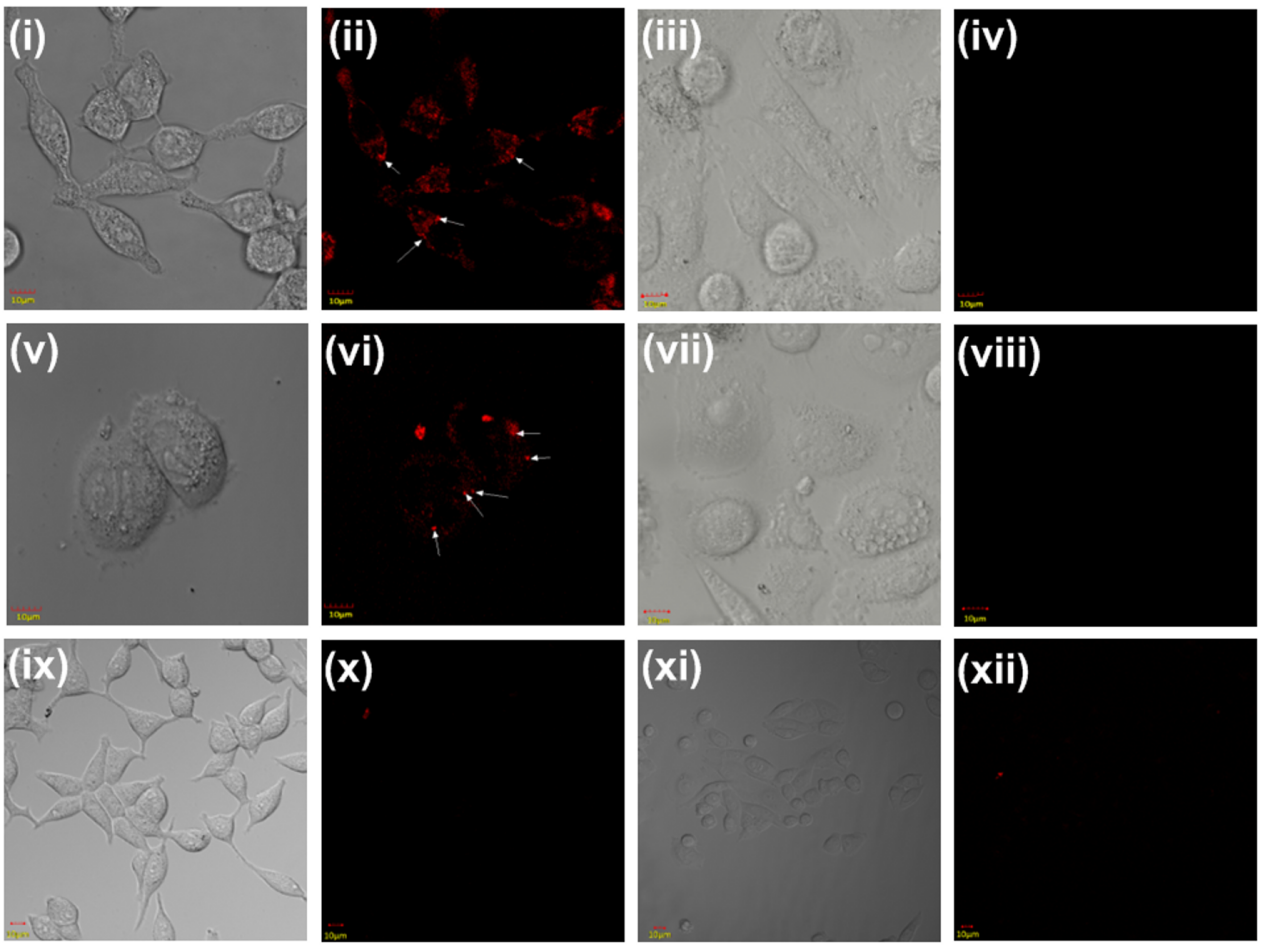
(i) and (ix) DIC image of LNCaP cells (PSMA^+^); (ii) binding and internalization of DUPA-rhodamine B conjugate **13** to LNCaP cells by confocal microscopy at 100 nM concentration [endosomes are marked with white arrows]; (iii) and (vii) DIC image of PC-3 cells (PSMA^−^ and FR^−^); (iv) specificity of DUPA-rhodamine-B conjugate **13** in PSMA^−^ cell line such as PC-3 cells; (v) and (xi) DIC image of cells CHO-β cells (FR^+^); (vi) binding and internalization of pteroate-rhodamine B conjugate **17** in CHO-β cells by confocal microscopy at 150 nM concentration [endosomes are marked with white arrows]; (viii) specificity of pteroate-rhodamine B conjugate **17** in FR^−^ cell line such as PC-3 cells (DIC = differential interference contrast); (x) binding and internalization of DUPA-rhodamine B conjugate **13** to LNCaP cells in the presence of 100-fold excess 2-PMPA; (xii) binding and internalization of pteroate-rhodamine B conjugate **17** to CHO-β cells in the presence of 100-fold excess folic acid.

The newly synthesized bioconjugates **13** and **17**, that can selectively target PSMA^+^ and FR^+^ cancers, were further evaluated by performing in vitro studies using laser scanning confocal microscopy on PSMA^+^ LNCaP cells, FR^+^ epithelial CHO-β cells and PSMA^−^, FR^−^ PC-3 cells (Figure 3). The negative cell line was used to prove the protein specificity of newly synthesized ligand targeted bioconjugates **13** and **17**. In Figure 3 the confocal microscopic images depict the delivery of conjugates to cells via receptor-mediated endocytosis negating any possibility of non-specific uptake. The specificity of bioconjugates is indispensable to prevent collateral damage and toxicity to healthy cells when the chelating core is tethered to deliver radionuclides or cytotoxic drugs. The confocal microscopic images in Figure 3, panel (ii) shows the uptake of chelating DUPA rhodamine B conjugate in LNCaP cells at 100 nM concentration, panel (vi) shows the uptake of chelating pteroate rhodamine B conjugate in CHO-β cells at 150 nM concentration. The specificity of the ligand conjugates was further established by studying the uptake of bioconjugates **13** and **17** in malignant cells which express neither PSMA nor folate receptors [panels (iv) and (viii)]. The absence of any rhodamine B bioconjugates **13** and **17** uptake in the cytoplasm of negative cell line, PC-3 cells, show that the bioconjugates are very specific which is an important criterion in targeted drug delivery systems for avoiding off-site toxicity. In vitro specificity of bioconjugates, **13** and **17** were further examined by prior incubation of LNCaP cells and CHO-β cells with 100-fold excess of 2-PMPA and folic acid to block PSMA and folate receptors, respectively. Receptor blocked LNCaP and CHO-β cells display minimal uptake of bioconjugates **13** and **17** [panels (x) and (xii)], confirming the specificity of the synthesized bioconjugates. Thus, we have developed a novel strategy to synthesize targeted fluorescent tagged bioconjugates by introducing differentially protected α- and ε-amino groups of lysine amino acid derivative, Fmoc-Lys-(Tfa)-OH. Thus, our primary goal of introducing all the four components in **13** and **17** viz., targeting ligand, peptidic spacer, fluorescent tag as well as chelating core in a continuous synthesis process in cost effective manner, was achieved in high yield, purity and free of any heavy metal usually employed in other synthesis that is detrimental for biological studies.

## Conclusion

In conclusion, we have developed a new synthetic strategy for assembling targeting ligand, peptidic spacer, a fluorescent tag and chelating core in a continuous process without isolation of intermediates during the bioconjugate synthesis. The synthesis is carried out from a relatively non-expensive and commercially available H-Cys(trt)-(2-Cltrt) resin. The mode of linking the fluorophore to the growing peptide chain using a lysine derivative such as Fmoc-Lys(Tfa)-OH containing differentially protected amino groups that are labile only under basic conditions was found to be crucial in synthesizing the conjugates. With this synthetic protocol we have synthesized chelating DUPA rhodamine B and pteroate rhodamine B conjugates for targeting malignant cells as well as inflammatory cells expressing PSMA and folate receptors. The in vitro uptake study has been performed using laser scanning confocal microscopy and the bioconjugates are found to be delivered specifically to cells expressing corresponding cell surface proteins. The small molecule targeted imaging probes prepared in this study are designed for diagnosis and deep tissue imaging of cancers and inflammatory diseases. Near infrared fluorophores containing a free or activated carboxylic group (e.g., IRDye 800CW NHS ester) can also be conjugated with the peptidic spacer using this methodology through amide coupling reaction. Moreover, the bioconjugates can be employed as potential theranostic tools by attaching macromolecules, cytotoxic warheads, radioactive tracers, nanomaterials etc., via the chelating core.

## Experimental

### Materials and methods

H-Cys-2-ClTrt resin, Fmoc amino acids and amide coupling agents, reagents and solvents used in solid-phase peptide synthesis (SPPS) as well as in chemical synthesis were purchased from Iris Biotech GmbH, Sigma-Aldrich, Merck and Spectrochem. Dry solvents were prepared by using drying agents and following usual methods. Peptide syntheses were carried out in sintered glass peptide vessels (Chemglass) by standard peptide coupling procedures. ^1^H and ^13^C NMR data were recorded using a Bruker AV 400 MHz NMR spectrometer with TMS (tetramethylsilane) as internal reference. Mass spectra were recorded on a Brukermicro TOF-Q II spectrometer in positive mode and negative mode electrospray ionization methods. Reactions were monitored by TLC using MERCK 60 F_254_ pre-coated silica gel plates and the products were visualized under UV light. The purity of ligand targeted rhodamine B peptide conjugates was confirmed by a Dionex HPLC-Ultimate 3000 instrument and peptide conjugates were purified through Büchi reveleris prep instrument using RP-PFP column (XSelect CSH Prep Fluorophenyl 5 µm OBD).

### Synthesis of targeting ligand

**Procedure for synthesis (*S*)-5-benzyl 1-*tert*-butyl 2-(3-((*S*)-1,5-di-*tert*-butoxy-1,5-dioxopentan-2-yl)ureido)pentanedioate (3):** Triphosgene (0.050 g, 0.169 mmol) was dissolved in 3 mL dry DCM and the solution was stirred at −50 °C under an inert atmosphere (Scheme 1). Bis(*tert*-butyl)-L-glutamate·HCl (**1**, 0.150 g, 0.507 mmol) dissolved in 2 mL of dry DCM was added to triphosgene solution at −50 °C and triethylamine (0.5657 mL, 4.056 mmol) was added dropwise to the reaction mixture. The reaction mixture was stirred for 1.5 h at −50 °C and stirred for another 1.5 h at room temperature for the generation of isocyanate intermediate **2**. Thereafter, a solution of L-glutamic-γ-benzyl-α-*tert*-butyl·HCl (0.159 g, 0.507 mmol) and triethylamine (0.14 mL, 1.014 mmol) in DCM was added to the reaction mixture and the progress of the reaction was monitored through TLC using an ethyl acetate and hexane (1:3) mixture as eluent. The reaction mixture was further stirred overnight at room temperature. After the completion of reaction, the reaction mixture was concentrated under reduced pressure, diluted with ethyl acetate, washed with water and brine. The organic layer was dried over anhydrous Na_2_SO_4_, filtered and the solvent was evaporated under reduced pressure to afford crude reaction mixture which was purified by column chromatography over 100–200 mesh silica gel using 25% ethyl acetate and hexane as eluent. The purified benzyl tris(*tert*-butoxy) protected DUPA precursor **3**.

**(*S*)-5-Benzyl 1-*tert*-butyl 2-(3-((*S*)-1,5-di-*tert*-butoxy-1,5-dioxopentan-2-yl)ureido)pentanedioate (3):** Yellowish gummy liquid (yield = 85%, 249 mg), *R*_f_ = 0.29 (EtOAc/hexane = 1:3); ^1^H NMR (400 MHz, CDCl_3_) δ 7.33 (m, 5H), 5.10–5.04 (m, 4H), 4.38–4.28 (m, 2H), 2.51–2.37 (m, 2H), 2.32–2.23 (m, 2H), 2.20–2.12 (m, 1H), 2.09–2.00 (m, 1H), 1.96–1.81 (m, 2H), 1.44 (s, 9H), 1.43 (s, 9H), 1.41 (s, 9H); ^13^C NMR (100 MHz, CDCl_3_) δ 172.9, 172.5, 172.0, 171.9, 156.8, 135.8, 128.5, 128.2, 82.1, 82.0, 80.5, 66.4, 53.1, 53.0, 31.5, 30.3, 28.4, 28.3, 28.1, 28.0; HRMS (ESI) *m*/*z*: [M + Na]^+^ calcd for C_30_H_46_N_2_O_9_, 601.3096; found, 601.3092.

**Procedure for debenzylation of benzyl tris(*tert-*butoxy)-protected DUPA precursor 3 to give (*S*)-5-(*tert*-butoxy)-4-(3-((*S*)-1,5-di-*tert*-butoxy-1,5-dioxopentan-2-yl)ureido)-5-oxopentanoic acid (4):** To a solution of benzyl tris(*tert-*butoxy) protected DUPA precursor **3** (0.250 g, 0.434 mmol) in dichloromethane (10 mL), 10 mol % Pd/C (40 mg) was added. The reaction mixture was hydrogenated under an atmosphere of H_2_ gas (1 atm) for 24 h at room temperature. After completion of the reaction, Pd/C was filtered off through a celite bed and washed with DCM (3 × 5 mL). The solvent was evaporated under reduced pressure and the crude product was purified through column chromatography (hexane/ethyl acetate = 50:50) to afford the tris(*tert*-butoxy)-protected DUPA precursor **4** which was further used for peptide coupling reaction in the solid-phase peptide synthesis.

**(*S*)-5-(*tert*-Butoxy)-4-(3-((*S*)-1,5-di-*tert*-butoxy-1,5-dioxopentan-2-yl)ureido)-5-oxopentanoic acid (4):** Colourless viscous liquid solidified on standing (yield = 80%, 169 mg), *R*_f_ = 0.48 (EtOAc/hexane = 1:1); ^1^H NMR (400 MHz, CDCl_3_) δ 5.01 (d, *J* = 7.76 Hz, 2H), 4.32 (ddd, *J* = 5.0, 5.26, 7.76 Hz, 2H), 2.37–2.21 (m, 4H), 2.10–2.01 (m, 2H), 1.89–1.80 (m, 2H), 1.45 (s, 9H), 1.42 (s, 18H); ^13^C NMR (100 MHz, CDCl_3_) δ 176.1, 173.1, 172.5, 171.9, 157.8, 82.5, 82.1, 80.6, 53.3, 53.0, 31.5, 30.3, 28.4, 28.1, 28.0, 27.9, 27.8; HRMS (ESI) *m*/*z*: [M + Na]^+^ calcd for C_23_H_40_N_2_O_9_, 511.2626; found, 511.2640.

### General procedure for solid-phase synthesis

#### Resin swelling

All the resins used in solid-phase peptide synthesis were swelled initially with 5 mL of DCM for 30 minutes by bubbling nitrogen and after draining DCM, the resin is swelled once again with 5 mL DMF thrice for 15 minutes each.

### General procedure for the Kaiser test

Few resin beads were taken in a test-tube and 2 drops of each of ninhydrin, phenol and 0.1% potassium cyanide solution were added to the test-tube and heated for 2 minutes at 110 °C in a sand bath. The presence of free amine groups was confirmed by the appearance of dark blue colored resin beads in the test tube. The test was performed after coupling of each amino acid by the aforementioned procedure.

### General procedure for NHFmoc deprotection

The Fmoc-amino group in the growing peptide chain was deprotected in each step using 20% piperidine in DMF (10 mL) by bubbling nitrogen for 10 minutes through the swelled resin beads. The procedure was repeated thrice (1 × 4 mL; 2 × 3 mL) to ensure complete deprotection of Fmoc protecting group.

### General procedure for peptide cleavage from resin beads

A mixture of 9.25 mL trifluoroacetic acid (TFA), 0.25 mL triisopropylsilane (TIPS), 0.25 mL EDT and 0.25 mL H_2_O was prepared and 5 mL of this cocktail solution was added to resin beads and nitrogen was bubbled through the solution for 30 minutes. The same procedure was repeated twice using 2.5 mL of cocktail solution. The collected mother liquor from cleavage was evaporated under reduced pressure and the concentrated viscous liquid was precipitated in ice cold diethyl ether. The precipitated product was dried under nitrogen atmosphere and utilized for further studies.

### General procedure for solid-phase peptide synthesis

**Synthesis of PSMA targeted DUPA rhodamine B conjugate 13, DUPA-NH-(CH_2_)_7_CO-Phe-Phe-NH-(CH_2_)_7_CO-Lys(rhodamineB)-Dap-Asp-Cys:** H-Cys-2-ClTrt resin (0.050 g, 0.031 mmol) was initially swelled in DCM (5 mL) followed by DMF (5 mL). *N*-Fmoc-Asp(O*t*-Bu)-OH (0.032 g, 0.078 mmol), PyBOP (0.040 g, 0.078 mmol) and DIPEA (0.135 mL, 0.78 mmol) in 0.5 mL DMF was added to the peptide vessel containing resin beads and the coupling reaction was continued for 6 h. The resin beads were washed with DMF (3 × 5 mL) followed by the washing with isopropanol (3 × 3 mL). Completion of the peptide coupling reaction was confirmed by performing the Kaiser test (KT). Then a solution of 20% piperidine in DMF (1 × 4 mL; 2 × 3 mL) was added to the peptide vessel to cleave NHFmoc protecting group. Resin beads were washed with DMF (3 × 3 mL) followed by isopropanol (3 × 3 mL) and the formation of free amine was confirmed by the Kaiser test. After the swelling of resin in DMF, Boc-DAP(Fmoc)-OH (0.033 g, 0.078 mmol), PyBOP (0.040 g, 0.078 mmol) and DIPEA (0.135 mL, 0.78 mmol) in 0.5 mL DMF was added to the resin beads and the same steps were followed as described above. A series of amino acids including of Fmoc-Lys(Tfa)-OH (0.036 g, 0.078 mmol), Fmoc-8-aminocaprylic acid (0.030 g, 0.078 mmol), Fmoc-Phe-OH (0.030 g, 0.078 mmol), Phe-OH (0.030 g, 0.078 mmol) followed by Fmoc-8-aminocaprylic acid (0.030 g, 0.078 mmol) were coupled to the growing peptide chain as described earlier. After deprotection of Fmoc groups, tris(*tert*-butyl) protected DUPA (0.023 g, 0.047 mmol), PyBOP (0.040 g, 0.078 mmol) and DIPEA (0.135 mL, 0.78 mmol) in 0.5 mL DMF was added to the resin beads and swelled for 6 h. The completion of reaction was confirmed by the Kaiser test. At last the trifluoroacetyl-protected amino group of lysine was cleaved by treatment with 2 M aqueous piperidine for 6–12 h at room temperature and the complete deprotection of Tfa group was confirmed by the Kaiser test. Rhodamine B (0.023 g, 0.047), PyBOP (0.040 g, 0.078 mmol) and DIPEA (0.135 mL, 0.78 mmol) in 0.5 mL DMF was added to the peptide vessel and swelled for 6 h at room temperature. The completion of rhodamine B coupling reaction was confirmed by the Kaiser test. Finally, the resin was cleaved using a cocktail solution as described earlier in the Experimental section. The crude fluorescent peptide conjugate was concentrated under reduced pressure to evaporate TFA and ice-cold ether was added to precipitate the DUPA rhodamine B conjugate **13** as bright red solid. The crude product **13** was purified through a Büchi reveleris prep instrument using RP-PFP preparative column (XSelect CSH Prep Fluorophenyl 5 µm OBD, 5 µm, 19 mm × 150 mm) at λ = 280 or 555 nm (detailed procedure was mentioned in the preparative HPLC chromatography method). Acetonitrile was removed under reduced pressure, and pure fractions were freeze-dried to yield DUPA rhodamine B conjugate **13** as red solid. The yield of **13** was 76% (41 mg) and the purity of the conjugate **13** is further confirmed by reverse phase analytical high-pressure liquid chromatography (RP-HPLC) *t*_R_ = 9.8 min. The molecular mass is determined by LCMS. HRMS (+ESI) calcd for [M − Cl]^+^ (C_89_H_121_N_14_O_21_S)^+^: 1753.8546; found, 1753.8557.

**General procedure for solid-phase peptide synthesis of pteroate rhodamine B conjugate 17, pteroate-NH-(CH_2_)_7_CO-Lys(rhodamine B)-DAP-Asp-Cys:** H-Cys(Trt)-2-ClTrt resin (0.050 g, 0.031 mmol) was swelled first using DCM (5 mL) followed by DMF (5 mL) according to the aforementioned procedure. Fmoc-Asp(O*t*-Bu)-OH (0.032g, 0.078 mmol), PyBOP (0.040 g, 0.078 mmol) and DIPEA (0.135 mL, 0.78 mmol) in 0.3 mL DMF was added to peptide vessel with resin beads and bubbled using nitrogen gas for 6 h. The resin beads were washed with DMF (3 × 5 mL) followed by the washing with isopropanol (3 × 3 mL). The completion of reaction was confirmed by performing the Kaiser test. After the completion of coupling, a solution of 20% piperidine in DMF (1 × 4 mL; 2 × 3 mL) was added to the peptide vessel to cleave the NHFmoc protecting group according to the procedure mentioned in experimental section. Resin beads were washed with DMF (3 × 3 mL) and isopropanol (3 × 3mL) and the formation of free amine was confirmed by the Kaiser test. After swelling the resin again in DMF, Boc-Dap(Fmoc)-OH (0.033 g, 0.078 mmol), PyBOP (0.040 g, 0.078 mmol) and DIPEA (0.135 mL, 0.78 mmol) in 0.5 mL DMF was added to the resin and same steps were followed as described above. Amino acids Fmoc-Lys(Tfa)-OH (0.036 g, 0.078 mmol), Fmoc-8-aminocaprylic acid (0.030g, 0.078 mmol) were coupled sequentially to the peptide chain following similar procedure. Finally after the cleavage of NHFmoc group from the peptide chain using 20% piperidine in DMF (1 × 4 mL; 2 × 3 mL), *N*^10^-(trifluoroacetyl)pteroic acid (0.046 g, 0.019 mmol), PyBOP (0.040 g, 0.078 mmol) and DIPEA (0.135 mL, 0.78 mmol) in 0.3 mL DMSO was added to the resin beads in peptide vessel and bubbled for 6 h. The peptide vessel was wrapped with aluminum foil to protect from light. The completion of reaction was ensured by the Kaiser test after washing the resin beads with DMF (3 × 5 mL) and isopropanol (3 × 5 mL). An aliquot of 1% hydrazine in DMF (3 × 2 mL) was added to the resin beads to deprotect *N*^10^-(trifluoroacetyl) protecting group by bubbling through resin beads for 10 min each. The resin beads were washed with DMF (3 × 3 mL) followed by isopropanol (3 × 3 mL). The secondary amine group generated in the pteroate core does not give a positive Kaiser test. The lysine trifluoroacetyl protected amino group was now deprotected by 6–12 h treatment with 2 M aqueous piperidine at room temperature and the completion of deprotection was confirmed by the Kaiser test. Rhodamine B (0.023 g, 0.047 mmol), PyBOP (0.040 g, 0.078 mmol) and DIPEA (0.135 mL, 0.78 mmol) in 0.5 mL DMF was added to the resin beads in the peptide vessel and the coupling was continued for 6 h at room temperature. The completion of the reaction was confirmed by the Kaiser test. The resin beads were dried for 30 minutes under nitrogen atmosphere. The pteroate rhodamine B conjugate **17** was obtained as a red precipitate after cleavage from the resin beads. The crude product **17** was purified through a Büchi reveleris prep instrument using RP-PFP (pentafluorophenyl) preparative column (5 µm, 19 mm × 150 mm) at λ = 280 or 555 nm (detailed procedure was mentioned in the preparative HPLC chromatography method). Acetonitrile was removed under reduced pressure, and pure fractions were freeze-dried to yield pteroate Rhodamine B conjugate **17** as red solid. The yield of the product **17** was 70% (33 mg) and the purity of the conjugate **17** is further confirmed by reverse phase analytical high-pressure liquid chromatography, (RP-HPLC) *t*_R_ = 3.09 min. The molecular mass is determined by LCMS and HRMS (+ESI) calcd for [M − Cl]^+^ (C_66_H_84_N_15_O_12_S)^+^: 1310.6139; found, 1310.6352.

### Analytical HPLC method

The purity of bioconjugates **13** and **17** were analyzed using a Dionex HPLC-Ultimate 3000 system. Typically a solution of either **13** or **17** (20 μL, 1.0 mg/1.0 mL) dissolved in a mixture of CH_3_CN/H_2_O (1:1) was injected via the autosampler and eluted using a Dionex Acclaim^®^ 120 C_18_, 5 μm, 4.6 mm × 250 mm analytical column at a flow rate of 1 mL/min (mobile phase, A = 0.1% trifluoro acetic acid/H_2_O and B = acetonitrile). An isocratic flow of 40% B (v/v) was used during the run for 0 to 4 min and gradually a linear gradient of B upto 100% B (v/v) was applied over a period of 40 min. The chromatogram was recorded using Ultimate 3000 RS Variable Wavelength detector at 225–280 nm.

### Preparative HPLC method

The purification of bioconjugates **13** and **17** was performed using a Büchi Reveleris Prep HPLC System. Crude bioconjugates **13** or **17** were dissolved in a mixture of CH_3_CN/H_2_O (1:1, 1 mL) and injected into the sample injector for elution using a RP-PFP (Reverse Phase PentafluoroPhenyl) preparative column (XSelect CSH Prep Fluorophenyl 5 µm OBD, 19 mm × 150 mm). A flow rate of 10 mL/min (mobile phase, A = 0.1% trifluoro acetic acid/H_2_O and B = acetonitrile) is maintained throughout the run and the mobile phase gradient was changed from 1% B (v/v) to 50% B (v/v) over a period of 40 min. The mobile phase gradient was further changed to 80% B (v/v) in the next 15 min and the chromatogram was recorded at λ = 280 or 555 nm. Pure fractions of **13** or **17** were collected using an automatic fraction collector, acetonitrile was evaporated under reduced pressure and freeze dried to obtain pure conjugates **13** or **17**.

**Culture of human cancer and epithelial cell lines:** LNCaP and CHO-β cells were obtained as gift from Prof. Philip S. Low, Purdue University, West Lafayette, USA whereas the PC-3 cell line was purchased from NCCS, Pune, India. LNCaP cells were grown as a monolayer using 1640 RPMI medium containing 10% heat-inactivated fetal bovine serum and 1% penicillin streptomycin and CHO-β cells in folate-deficient RPMI 1640 containing 10% heat-inactivated foetal bovine serum and supplemented with 1% penicillin streptomycin. Both the cell lines were grown in a 5% CO_2_:95% air-humidified atmosphere at 37 °C.

**Procedure for uptake study of peptide conjugates 13 and 17 using laser scanning confocal microscope in human cancer cell lines LNCaP, PC-3 and epithelial cell line CHO-β:** LNCaP cells (15,000 cells/well in 1 mL), CHO-β (10,000 cells/well in 1 mL) and PC-3 (10,000 cells/well) were seeded into German borosilicate confocal dishes and allowed cells to form monolayers over 24 h. Spent medium was replaced with fresh medium containing DUPA-rhodamine B, **13** (100 nM) and pteroate-rhodamine B, **17** (150 nM) in LNCaP and CHO-β cells, respectively and the cells were incubated with the compound for 1 h at 37 °C. For competition experiments, a 100-fold excess concentration of binding ligand, 2-PMPA for LNCap cells and folic acid for CHO-β cells were incubated for 1 h at 37 °C prior to incubation with bioconjugate **13** (100 nM) and **17** (150 nM), respectively. After rinsing with fresh medium (3 × 1.0 mL) to remove unbound conjugates, confocal images were acquired using a laser scanning confocal microscopy (FV 1000, Olympus) by excitation at 559 nm (yellow diode laser) and emission at 618 nm.

## Author’s Contributions

Sagnik Sengupta (SS) chemically synthesized and characterized the targeting ligands and bioconjugate **13**, Bishnubasu Giri synthesized and characterized the bioconjugate **17**, Mena Asha Krishnan (MAK) performed biological studies of bioconjugates **13** and **17** using confocal microscopy on various cell lines, SS, MAK, Premansh Dudhe and Ramesh B Reddy wrote the initial draft of the manuscript and involved in updating the latest references with proper format. Sudeshna Chattopadhyay and Venkatesh Chelvam ideated the research, executed the project for biological and nanomaterial delivery applications along with drafting and revising the final manuscript critically for important intellectual content.

## Supporting Information

File 1 NMR and HRMS spectra of compounds **3**, **4** and analytical HPLC spectra of purified (λ at 254 nm and 225 nm) as well as crude (λ at 225 nm), electrospray ionization mass spectra and high-resolution mass spectra for bioconjugates **13** and **17**.
