# Supplementary material for: Novel solid-phase strategy for the synthesis of ligand-targeted fluorescent-labelled chelating peptide conjugates as a theranostic tool for cancer
Source: Beilstein J Org Chem. 2018 Oct 18;14:2665–79. doi: 10.3762/bjoc.14.244 (PMC6204756; doi:10.3762/bjoc.14.244)
Supplement: File 1 — NMR and HRMS spectra of compounds 3, 4 and analytical HPLC spectra of purified (λ at 254 nm and 225 nm) as well as crude (λ at 225 nm), electrospray ionization mass spectra and high-resolution mass spectra for bioconjugates 13 and 17. [file Beilstein_J_Org_Chem-14-2665-s001.pdf]

Supporting Information

for

Novel solid-phase strategy for the synthesis of ligand-argeted fluorescent-labelled chelating peptide conjugates as a theranostic tool for cancer

Sagnik Sengupta[1], Mena Asha Krishnan[2], Premansh Dudhe[1], Ramesh B. Reddy[1], Bishnubasu Giri[1], Sudeshna Chattopadhyay[2,3] and Venkatesh Chelvam*,§,1,2

Address: [1]Discipline of Chemistry, Indian Institute of Technology Indore, Khandwa Road, Simrol, Indore 453 552, India, [2]Discipline of Biosciences and Biomedical Engineering, Indian Institute of Technology Indore, Khandwa Road, Simrol, Indore 453 552, India and [3] Discipline of Physics and Discipline of Metallurgy Engineering & Material Sciences, Indian Institute of Technology Indore, Khandwa Road, Simrol, Indore 453 552, India.

Email: Venkatesh Chelvam: cvenkat@iiti.ac.in;
§Phone: +91-731-2438789

*Corresponding author

NMR and HRMS spectra of compounds 3, 4 and analytical HPLC spectra of purified (λ at 254 nm and 225 nm) as well as crude (λ at 225 nm), electrospray ionization mass spectra and high-resolution mass spectra for bioconjugates 13 and 17.

Table of contents

**Figure S1**	High-resolution mass spectrometry data for PSMA targeting DUPA rhodamine B chelating conjugate **13**
**Figure S2**	High-resolution mass spectrometry data pteroate rhodamine B chelating conjugate **17**
**Figure S3**	ESIMS spectra for PSMA targeting DUPA rhodamine B chelating conjugate **13**
**Figure S4**	ESIMS spectra for pteroate rhodamine B chelating conjugate **17**
**Figure S5**	Analytical LC data of DUPA rhodamine B chelating conjugate **13** (Abs. at 254 nm)
**Figure S6**	Analytical LC data of pteroate rhodamine B chelating conjugate **17 (**Abs. at 254 nm)
**Figure S7**	Analytical LC data of crude DUPA rhodamine B chelating conjugate **13** (Abs. at 225 nm)
**Figure S8**	Analytical LC data of DUPA rhodamine B chelating conjugate **13** (Abs. at 225 nm)
**Figure S9**	Analytical LC data of crude pteroate rhodamine B chelating conjugate **17** (Abs. At 225 nm)
**Figure S10**	Analytical LC data of pteroate rhodamine B chelating conjugate **17 (**Abs. at 225 nm)
**Figure S11**	^{1}H NMR of (*S*)-5-benzyl 1-*tert*-butyl 2-(3-((*S*)-1,5-di-*tert*-butoxy-1,5-dioxopentan-2-yl)ureido)pentanedioate (**3**)
**Figure S12**	^{13}C NMR of *S*)-5-benzyl 1-*tert*-butyl 2-(3-((*S*)-1,5-di-*tert*-butoxy-1,5-dioxopentan-2-yl)ureido)pentanedioate (**3**)
**Figure S13**	HRMS spectra of *S*)-5-benzyl 1-*tert*-butyl 2-(3-((*S*)-1,5-di-*tert*-butoxy-1,5-dioxopentan-2-yl)ureido)pentanedioate (**3**)
**Figure S14**	^{1}H NMR of (*S*)-5-(*tert*-butoxy)-4-(3-((*S*)-1,5-di-*tert*-butoxy-1,5-dioxopentan-2-yl)ureido)-5-oxopentanoic acid (**4**)
**Figure S15**	^{13}C NMR of (*S*)-5-(*tert*-butoxy)-4-(3-((*S*)-1,5-di-*tert*-butoxy-1,5-dioxopentan-2-yl)ureido)-5-oxopentanoic acid (**4**)
**Figure S16**	HRMS spectra (*S*)-5-(*tert*-butoxy)-4-(3-((*S*)-1,5-di-*tert*-butoxy-1,5-dioxopentan-2-yl)ureido)-5-oxopentanoic acid (**4**)

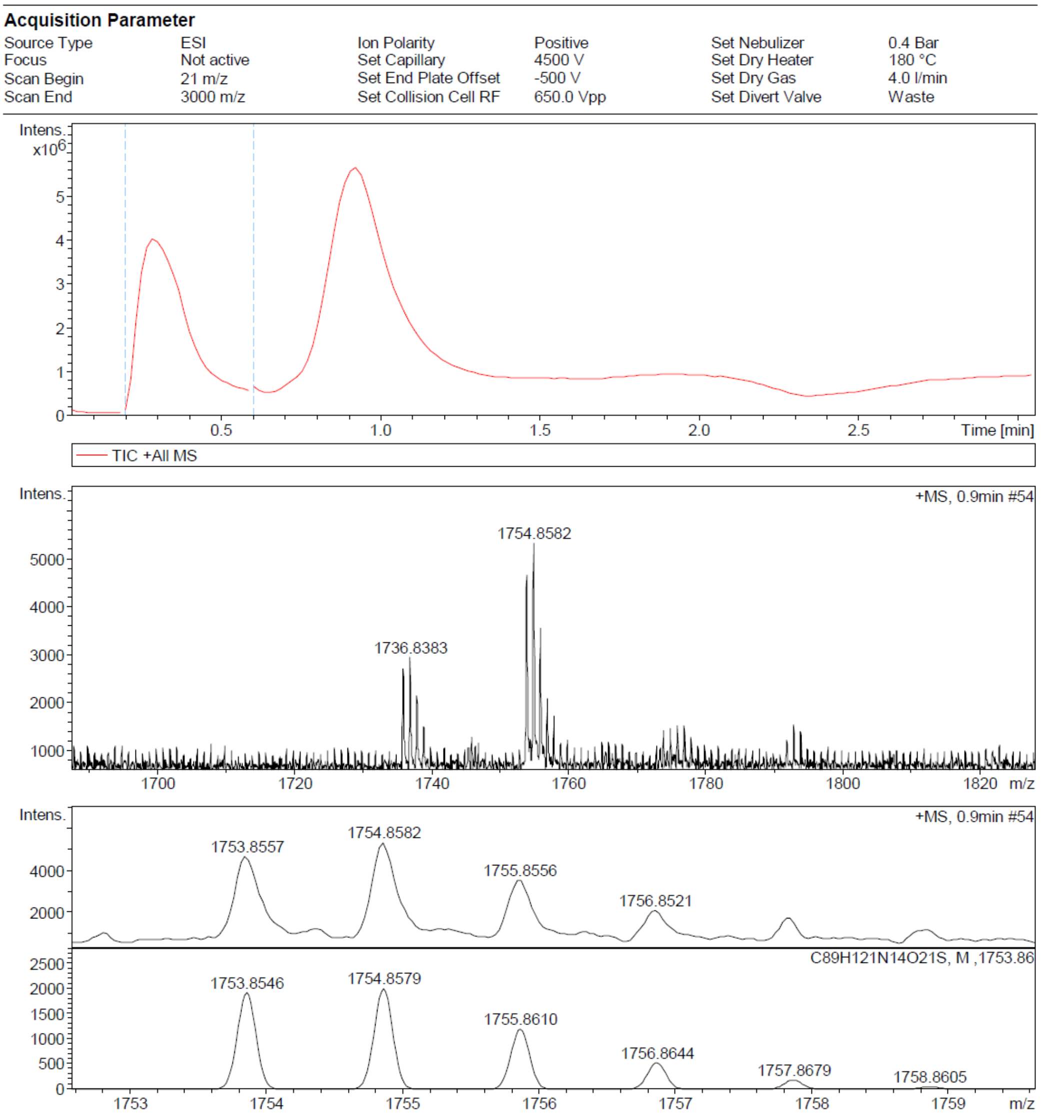

Figure S1: High-resolution mass spectrometry data for PSMA targeting DUPA rhodamine B chelating conjugate **13**.

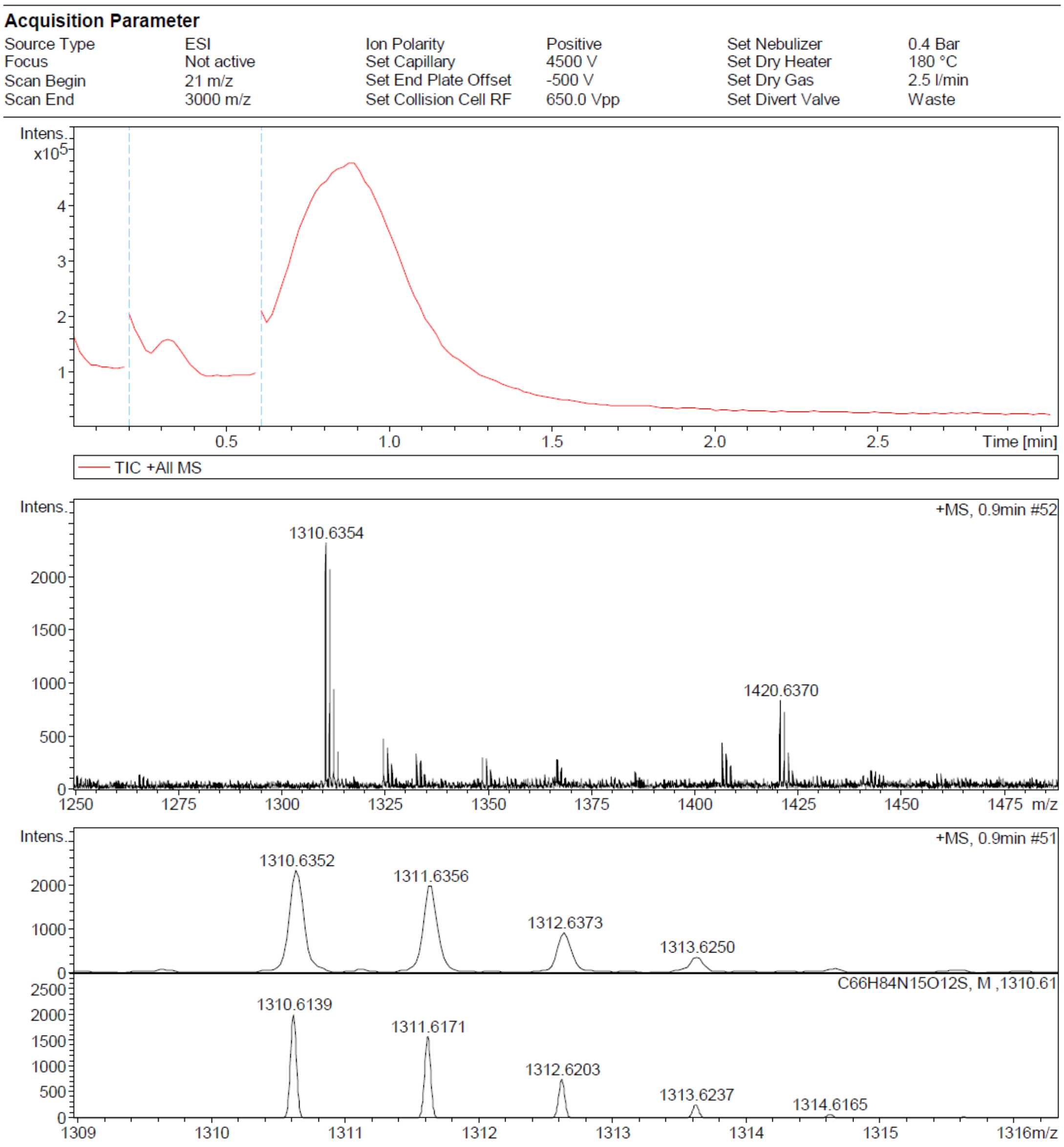

Figure S2: High-resolution mass spectrometry data pteroate rhodamine B chelating conjugate **17**.

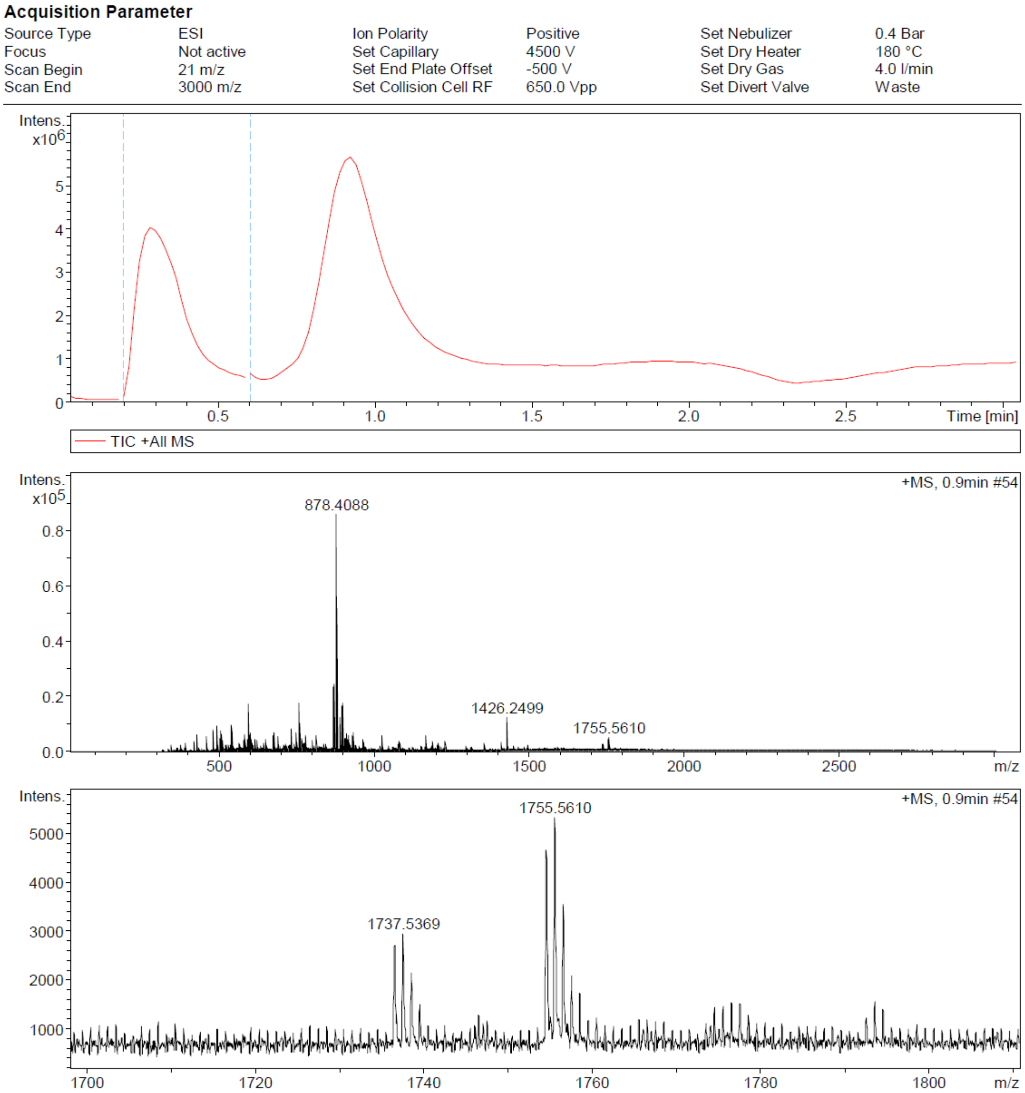

Figure S3: ESIMS spectra for PSMA targeting DUPA rhodamine B chelating conjugate **13**.

Acquisition Parameter

Source Type	ESI	Ion Polarity	Positive	Set Nebulizer	0.4 Bar
Focus	Not active	Set Capillary	4500 V	Set Dry Heater	180 °C
Scan Begin	21 m/z	Set End Plate Offset	-500 V	Set Dry Gas	2.5 l/min
Scan End	3000 m/z	Set Collision Cell RF	650.0 Vpp	Set Divert Valve	Waste

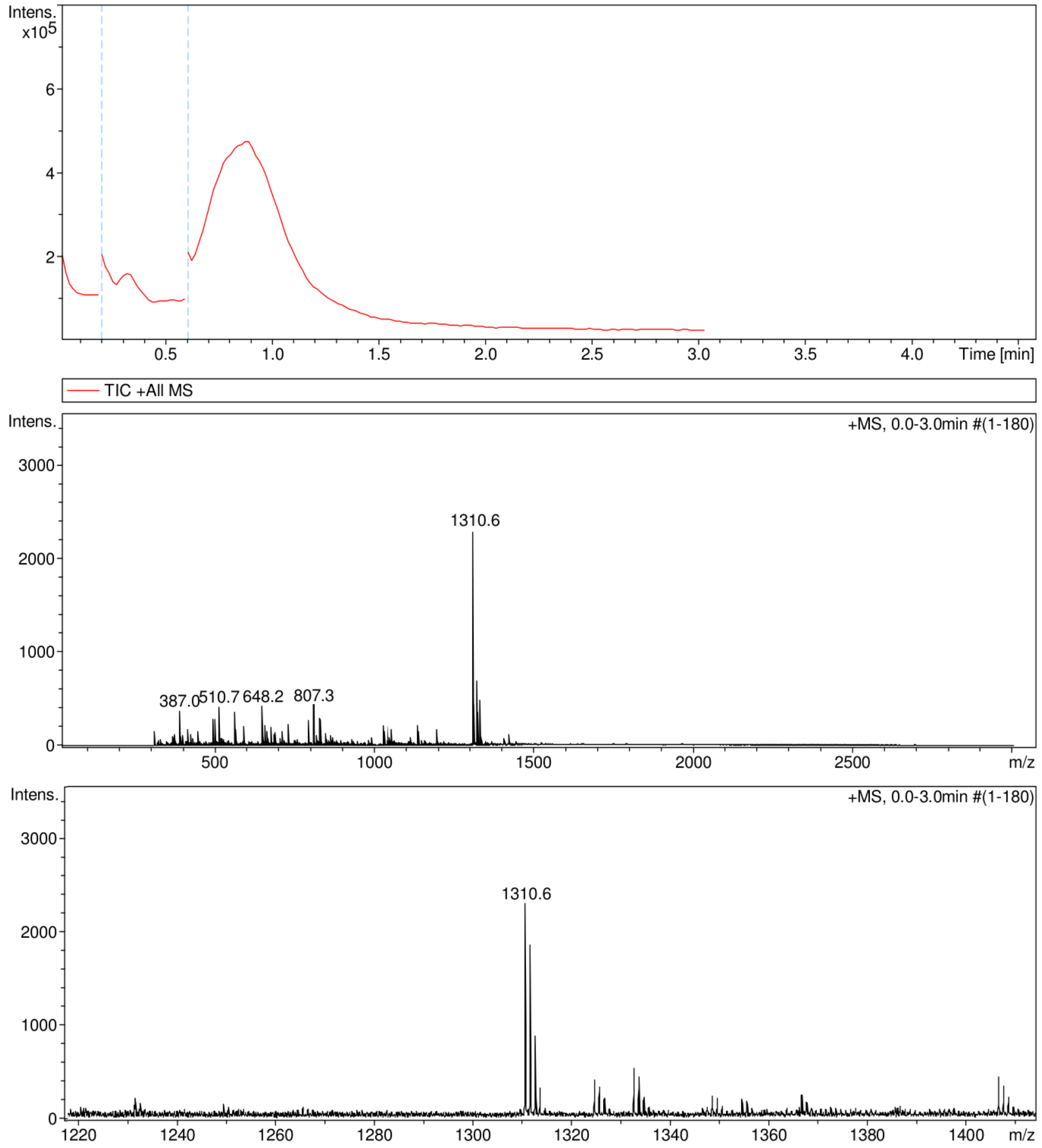

Figure S4: ESIMS spectra for pteroate rhodamine B chelating conjugate **17**.

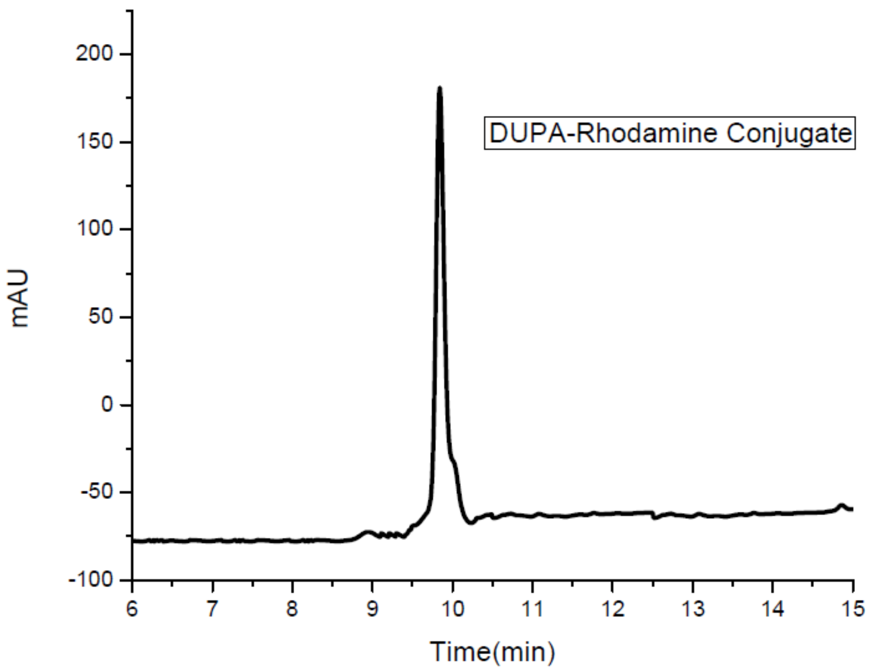

Figure S5: Analytical LC data of DUPA rhodamine B chelating conjugate **13** (Abs. at 254 nm).

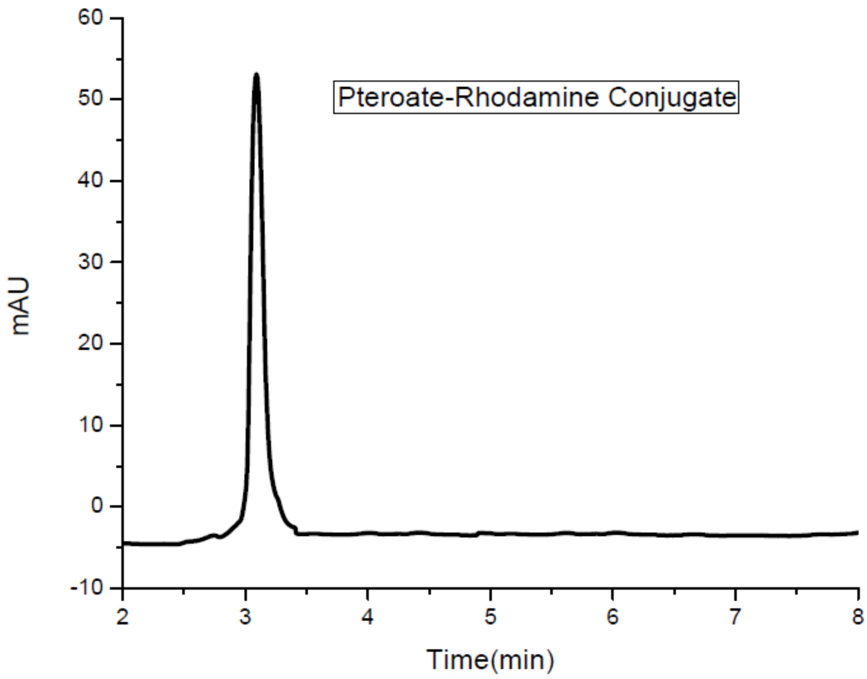

Figure S6: Analytical LC data of pteroate rhodamine B chelating conjugate **17** (Abs. at 254 nm).

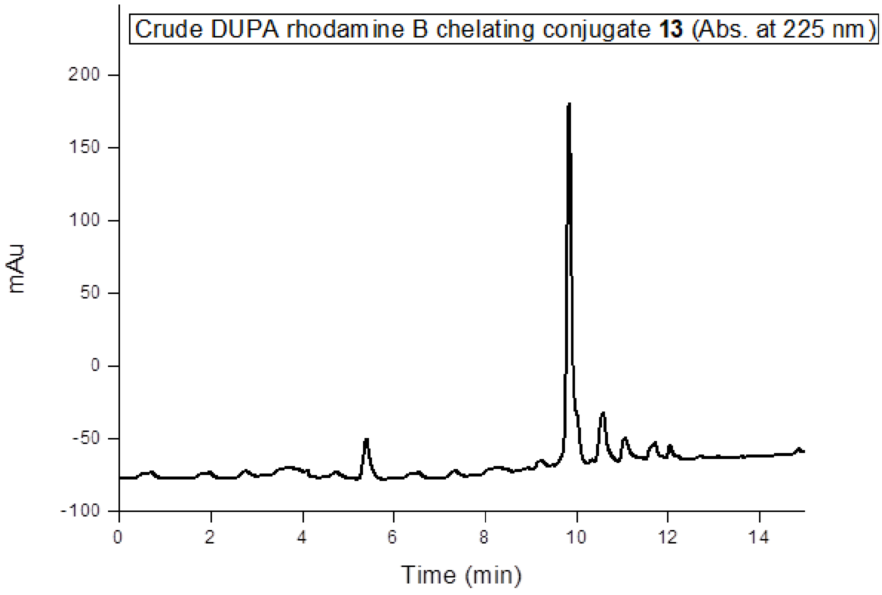

Figure S7: Analytical LC data of crude DUPA rhodamine B chelating conjugate **13** (Abs.at 225 nm).

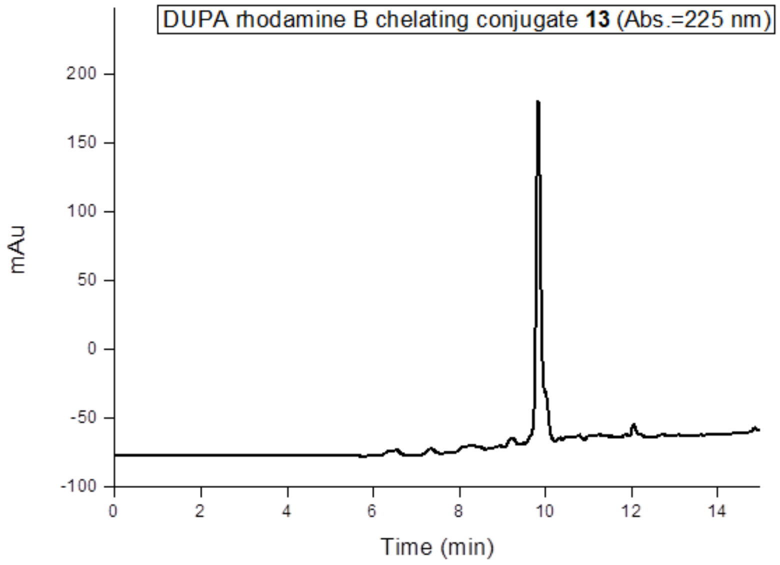

Figure S8: Analytical LC data of purified DUPA rhodamine B chelating conjugate **13** (Abs. at 225 nm).

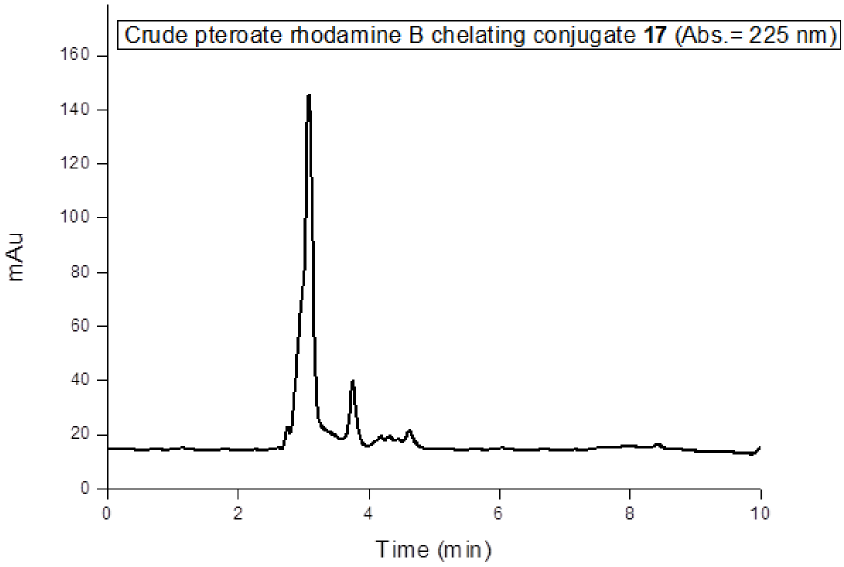

Figure S9: Analytical LC data of crude pteroate rhodamine B chelating conjugate **17** (Abs.at 225 nm).

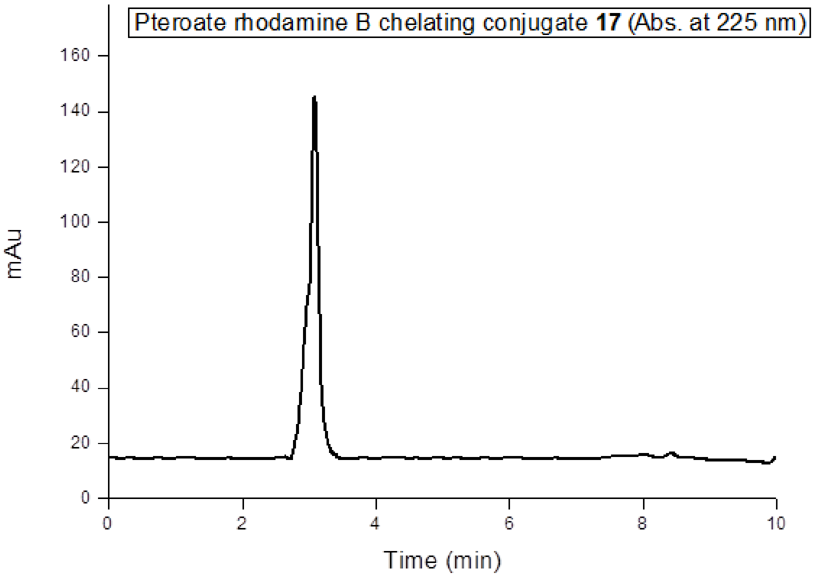

Figure S10:. Analytical LC data of purified pteroate rhodamine B chelating conjugate **17** (Abs. at 225 nm).

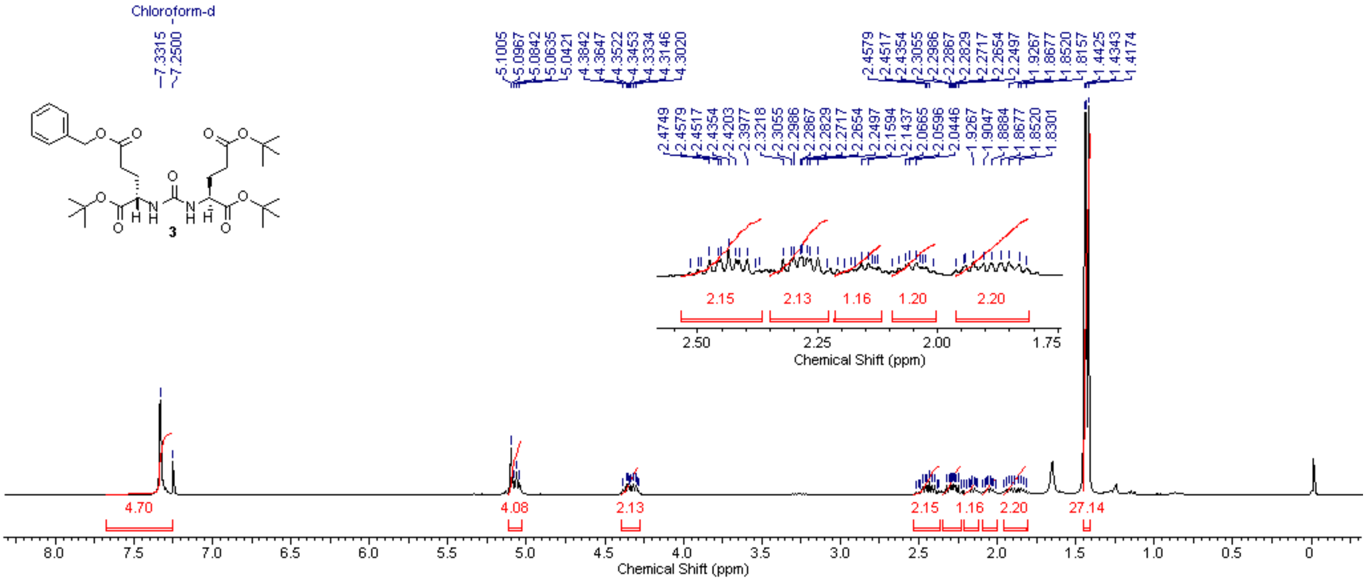

Figure S11: ^{1}H NMR of (*S*)-5-benzyl 1-*tert*-butyl 2-(3-((*S*)-1,5-di-*tert*-butoxy-1,5-dioxopentan-2-yl)ureido)pentanedioate (**3**).

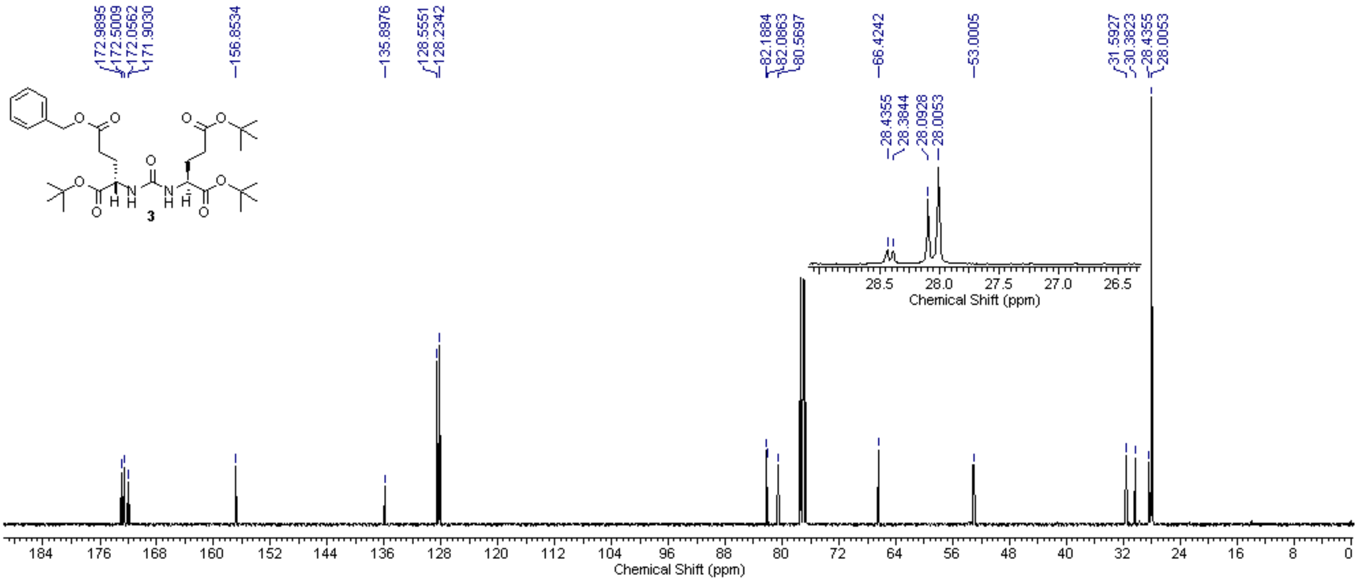

Figure S12: ^{13}C NMR of (*S*)-5-benzyl 1-*tert*-butyl 2-(3-((*S*)-1,5-di-*tert*-butoxy-1,5-dioxopentan-2-yl)ureido)pentanedioate (**3**).

Acquisition Parameter

Source Type	ESI	Ion Polarity	Positive	Set Nebulizer	0.4 Bar
Focus	Active	Set Capillary	4500 V	Set Dry Heater	180 °C
Scan Begin	50 m/z	Set End Plate Offset	-500 V	Set Dry Gas	4.0 l/min
Scan End	3000 m/z	Set Collision Cell RF	650.0 Vpp	Set Divert Valve	Waste

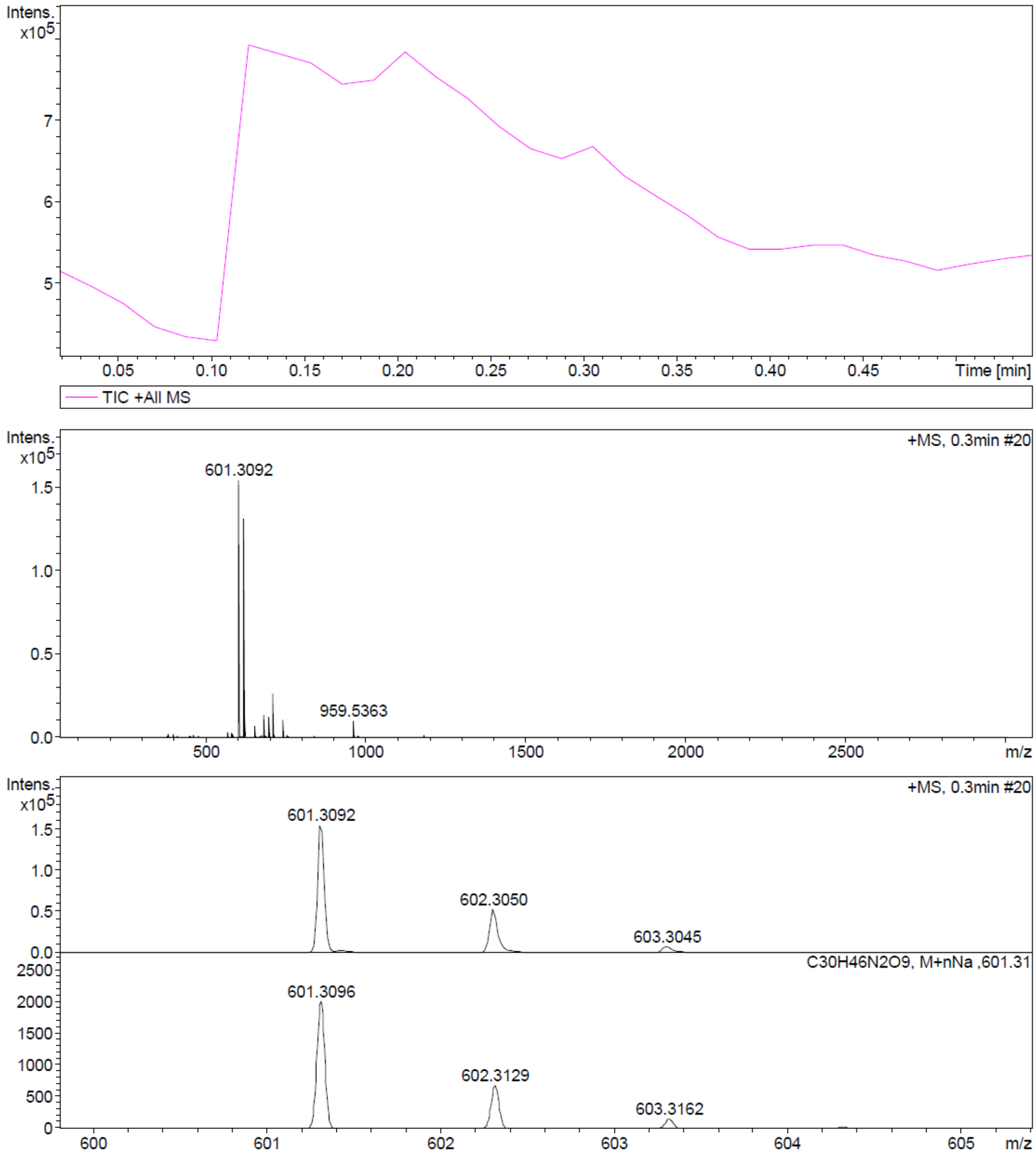

Figure S13: HRMS spectra of (*S*)-5-benzyl 1-*tert*-butyl 2-(3-((*S*)-1,5-di-*tert*-butoxy-1,5-dioxopentan-2-yl)ureido)pentanedioate (**3**).

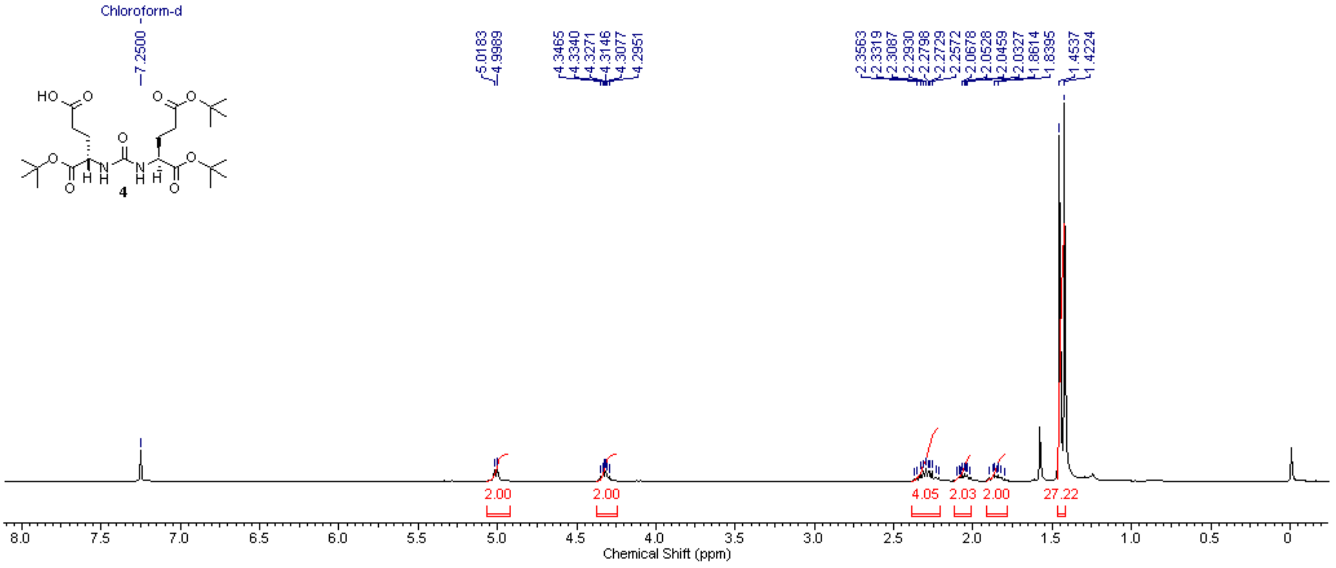

Figure S14: ^{1}H NMR of (*S*)-5-(*tert*-butoxy)-4-(3-((*S*)-1,5-di-*tert*-butoxy-1,5-dioxopentan-2-yl)ureido)-5-oxopentanoic acid (**4**).

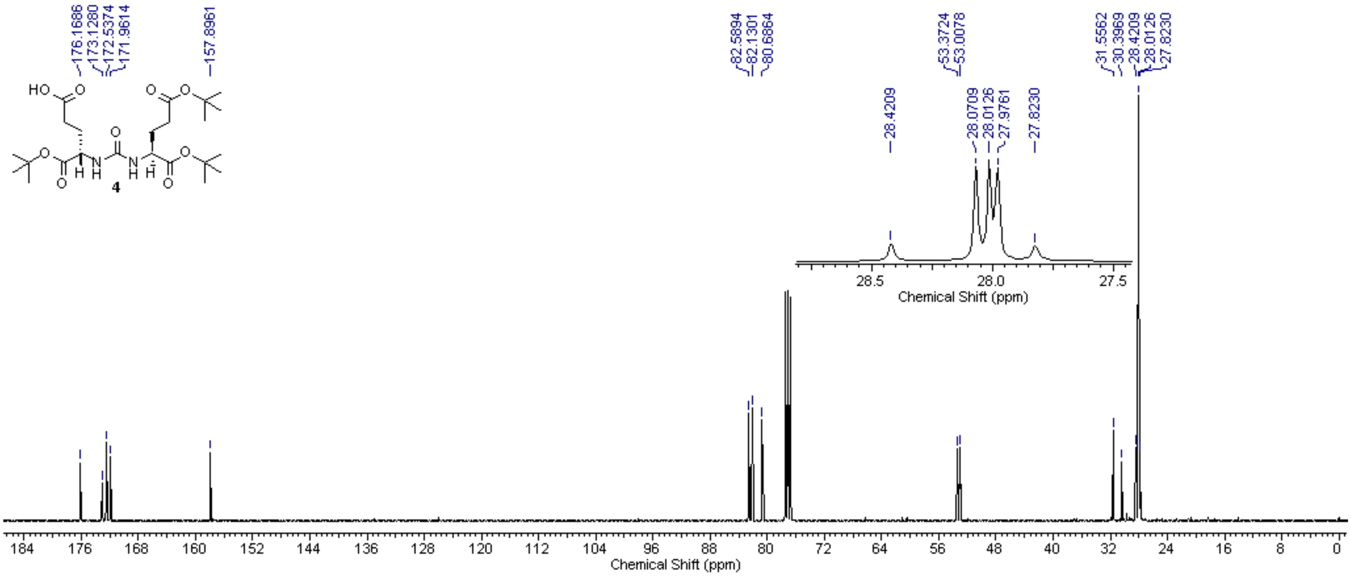

Figure S15: ^{13}C NMR of (*S*)-5-(*tert*-butoxy)-4-(3-((*S*)-1,5-di-*tert*-butoxy-1,5-dioxopentan-2-yl)ureido)-5-oxopentanoic acid (**4**).

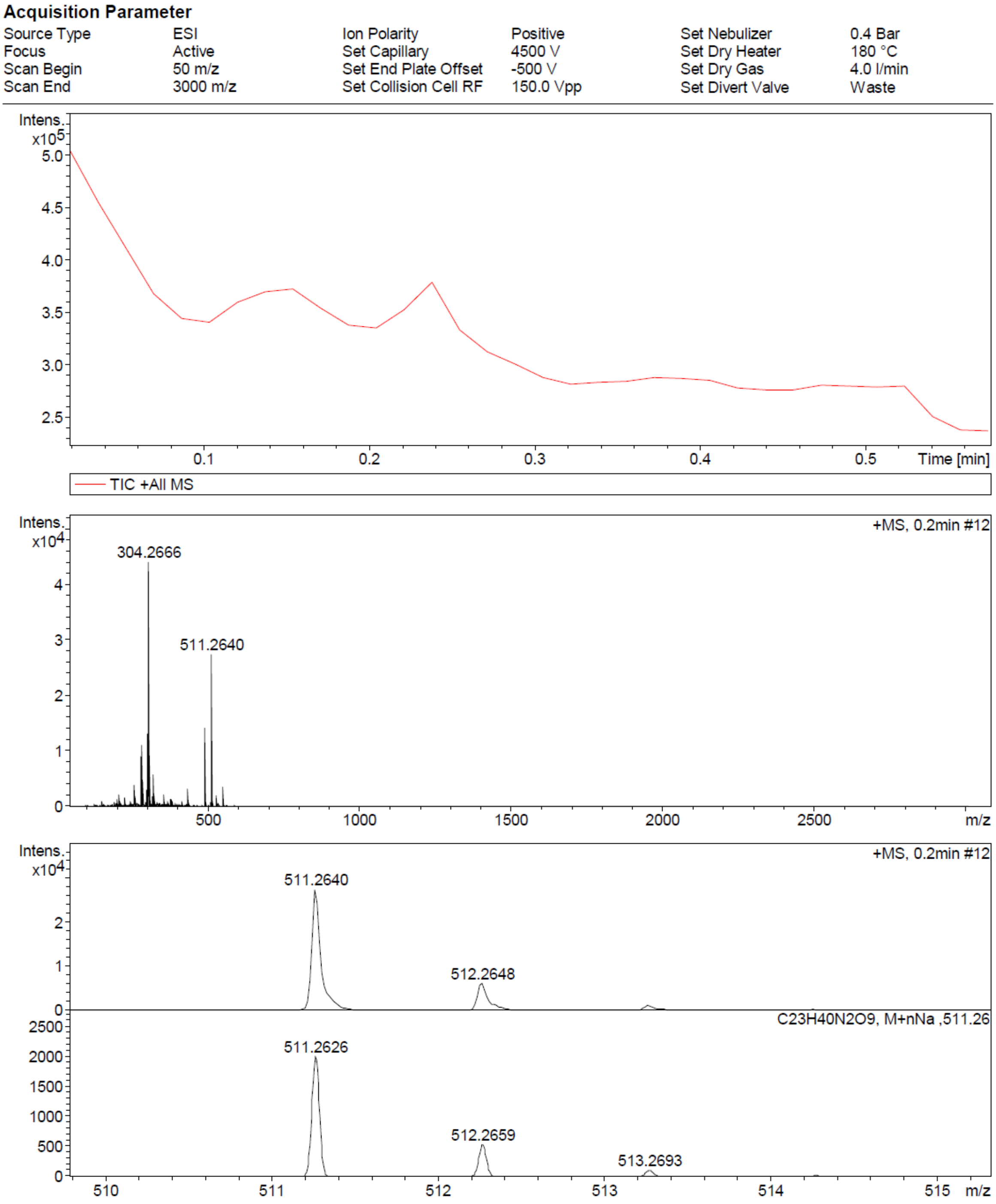

Figure S16. HRMS of (*S*)-5-(*tert*-butoxy)-4-(3-((*S*)-1,5-di-*tert*-butoxy-1,5-dioxopentan-2-yl)ureido)-5-oxopentanoic acid (**4**).
